# TM4SF5-mediated abnormal food-intake behavior and apelin expression facilitate non-alcoholic fatty liver disease features

**DOI:** 10.1016/j.isci.2023.107625

**Published:** 2023-08-14

**Authors:** Yangie Dwi Pinanga, Han Ah Lee, Eun-Ae Shin, Haesong Lee, Kyung-hee Pyo, Ji Eon Kim, Eun Hae Lee, Wonsik Kim, Soyeon Kim, Hwi Young Kim, Jung Weon Lee

**Affiliations:** 1 Department of Pharmacy, College of Pharmacy, Seoul National University, Seoul 08826, Republic of Korea; 2 Department of Internal Medicine, Ewha Womans University College of Medicine, Division of Gastroenterology and Hepatology, Ewha Womans University Mokdong Hospital, Seoul 07985, Republic of Korea; 3 Research Institute of Pharmaceutical Sciences, College of Pharmacy, Seoul National University, Seoul 08826, Republic of Korea

**Keywords:** Biological sciences, Cell biology, Molecular biology, Physiology

## Abstract

Transmembrane 4 L six family member 5 (TM4SF5) engages in non-alcoholic steatohepatitis (NASH), although its mechanistic roles are unclear. Genetically engineered *Tm4sf5* mice fed *ad libitum* normal chow or high-fat diet for either an entire day or a daytime-feeding (DF) pattern were analyzed for metabolic parameters. Compared to wild-type and *Tm4sf5*^−/−^ knockout mice, hepatocyte-specific TM4SF5-overexpressing *Alb*-TG^Tm4sf5−Flag^ (TG) mice showed abnormal food-intake behavior during the mouse-inactive daytime, increased apelin expression, increased food intake, and higher levels of NASH features. DF or exogenous apelin injection of TG mice caused severe hepatic pathology. TM4SF5-mediated abnormal food intake was correlated with peroxisomal β-oxidation, mTOR activation, and autophagy inhibition, with triggering NASH phenotypes. Non-alcoholic fatty liver disease (NAFLD) patients’ samples revealed a correlation between serum apelin and NAFLD activity score. Altogether, these observations suggest that hepatic TM4SF5 may cause abnormal food-intake behaviors to trigger steatohepatitic features via the regulation of peroxisomal β-oxidation, mTOR, and autophagy.

## Highlights

• Hepatic TM4SF5 appears to cause steatohepatitis, though unclear how to trigger it
• Hepatocyte-specific TM4SF5 expression caused abnormal food-intake and apelin level
• TM4SF5 triggered earlier steatohepatitic features via peroxisomal FAO at short HFD
• Modulation of TM4SF5-mediated apelin levels may be another strategy to avoid NASH

## Graphical abstract

**Figure:**
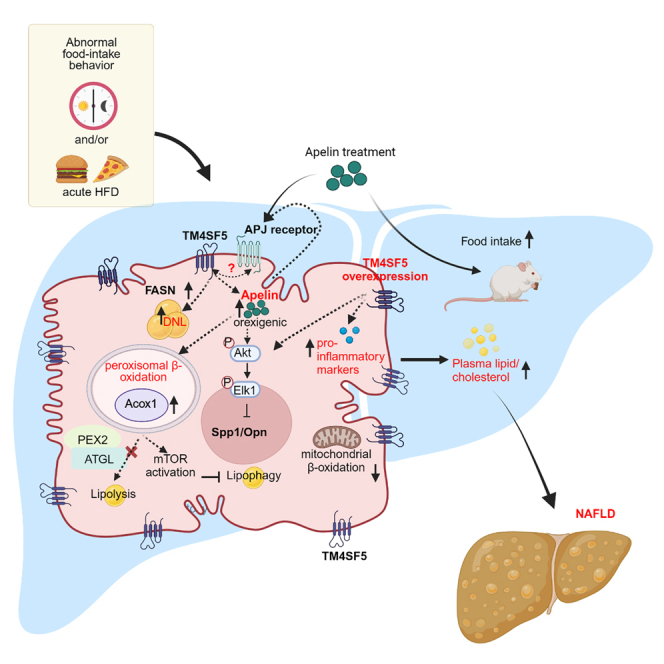

## Introduction

Non-alcoholic fatty liver disease (NAFLD) has reached 30% of the world’s population.^1^ Hepatitis C virus infections and metabolic dysfunctions can be considerable risk factors for NAFLD.^2^^,^^3^ Especially these days, western diets and abnormal diet patterns can lead to obesity and NAFLD, including non-alcoholic steatohepatitis (NASH).^4^ Abnormal eating habits and patterns, such as skipping breakfast and eating late at night, are associated with an increased risk of weight gain and adiposity, which can lead to obesity.^5^^,^^6^ In addition, diverse central and peripheral hormones, including peptides and steroids, influence appetite and food-intake behaviors through their actions on the hypothalamus, the brainstem, and the autonomic system.^7^ The hormones are secreted from adipose tissue, the pancreas, the brain, and the gastrointestinal tract,^8^^,^^9^ and they include leptin, adiponectin, apelin, fibroblast growth factor 21 (FGF21), osteopontin (SPP-1/OPN), neuropeptide Y (NPY), brain-derived neurotrophic factor (BDNF), ghrelin, cholecystokinin (CCK), glucagon-like peptide (GLP-1), insulin, and glucagon.^10^^,^^11^ The hormones can be either orexigenic, promoting food intake (e.g., ghrelin, glucagon, adiponectin, FGF21, NPY), or anorexigenic, suppressing food intake (e.g., GLP-1, insulin, leptin, growth differentiation factor 15 [GDF15], BDNF). In particular, liver-derived hormones that influence food-intake behaviors include liver-produced antimicrobial peptide 2 (LEAP2), FGF21, GDF15, apelin, and SPP-1/OPN. Among these hormones, the effect of apelin, which is also produced by adipose tissue as an adipokine, on eating behavior has been inconsistent, though apelin has potential roles in homeostasis, body fluid management, cell proliferation, and energy metabolism.^12^ In clinical and experimental studies, serum apelin levels are increased in obesity and insulin-resistant status,^13^ whereas apelin deficiency increases adiposity and blood fatty acid levels,^14^ and apelin overexpression is resistant to obesity.^15^ Furthermore, understanding how apelin and other hormones control food-intake behaviors is of great interest because abnormal metabolism-mediated pathological symptoms such as NASH, type 2 diabetes, cardiac dysfunction, and cancer can be fatal. Furthermore, the prevention of these diseases may be achieved via well-organized food-intake behaviors, voluntary exercise, and taking hormones to control appetite,^6^ in addition to therapeutically targeting cues to control metabolic activities.^16^ Identification of molecular cues that regulate food-intake behaviors and metabolic activity can thus be clinically beneficial for developing therapeutic reagents against abnormal metabolism-based diseases.

Transmembrane 4 L six family member 5 (TM4SF5) is a tetraspan(in) that consists of four transmembrane domains, two extracellular loops, an intracellular loop, and cytosolic N- and C-terminal tails.^17^ TM4SF5 is an *N*-glycosylated and palmitoylated protein that can be localized to plasma membranes,^18^ lysosomal membranes,^19^ and/or small extracellular vesicles.^20^ TM4SF5 is involved in the development of NAFLD^21^ and portal hypertension.^22^ Bidirectional TM4SF5-dependent crosstalks between hepatocytes and macrophages lead to the polarization and reprogramming of macrophages in chronic inflammatory environments to develop NASH-associated fibrosis.^23^ Under high-fat, carbohydrate, or fructose diets, TM4SF5-overexpressing mice show obesity and NASH-like phenotypes compared to TM4SF5-knockout mice.^20^^,^^21^^,^^24^ In addition, TM4SF5 is involved in blood glucose clearance^20^ and activation of the mammalian target of rapamycin (mTOR)/S6K1 pathway for protein translational processes.^19^ Therefore, TM4SF5-mediated changes in eating behaviors or appetite may be involved in abnormal metabolic dysfunctions, although whether and how TM4SF5 may do so during the earlier stages of NAFLD development have not been explored.

Here we investigated the mechanistic aspects of how TM4SF5 regulates food-intake behaviors and appetite hormone expression, leading to NAFLD features. We used TM4SF5-overexpressing or knockout (KO) mice fed normal chow diet (NCD) or high fat diet (HFD) *ad libitum* either over an entire day or during the daytime only for 1 or 2 weeks with or without intraperitoneal injection of apelin-13. We observed that hepatic TM4SF5 overexpression caused more food intake during the daytime, increased apelin production, and higher plasma and hepatic lipid levels, eventually leading to peroxisomal β-oxidation, mTOR activation, and autophagy inhibition, which are processes that may contribute to the development of earlier phenotypes for NASH.

## Results

### Hepatocyte-specific TG^Tm4sf5−Flag^ mice show abnormal food-intake behavior and increased apelin levels

**Figure 1:**
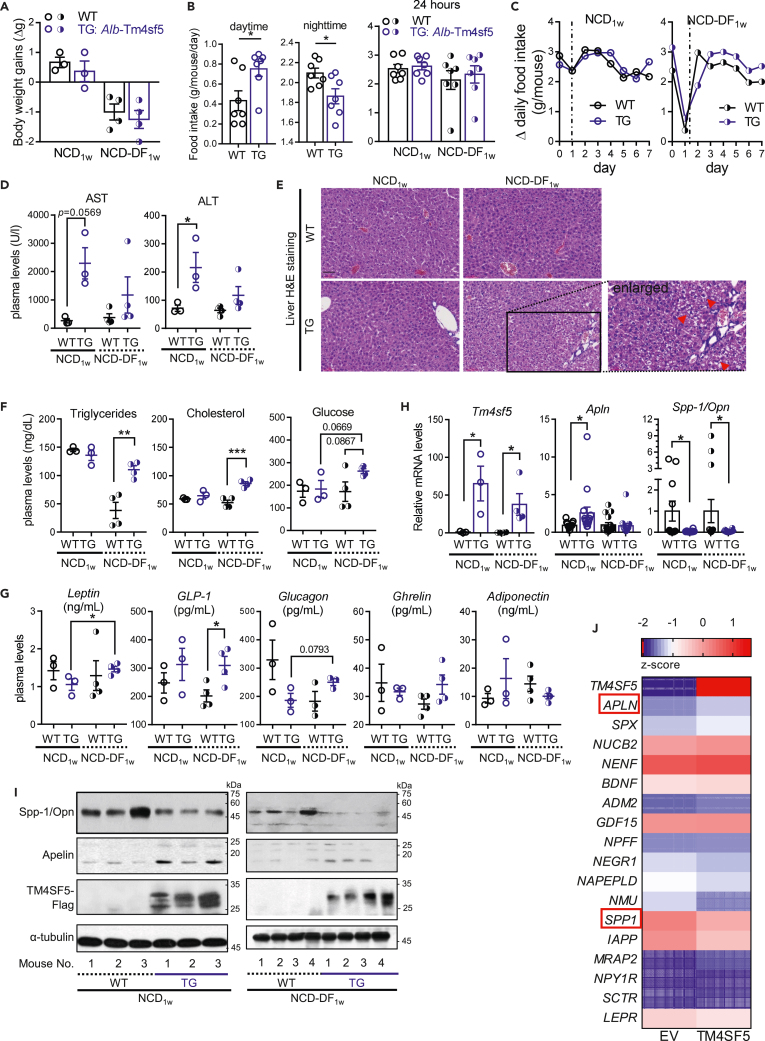
Hepatocyte-specific TG^Tm4sf5-Flag^ mice show abnormal food-intake behavior and increased apelin levels Nine-week-old male C57BL/6 mice (WT or TG *Alb*-TG^Tm4sf5−Flag^, n = 4) were fed normal chow *ad libitum* for an entire day (NCD_1w_) or during the daytime (9:00 a.m. to 9:00 p.m., NCD-DF_1w_) for 7 days before analyses. (A) Body weight changes (BWG, Δg). (B) Daytime or nighttime food intakes (g/mouse/day) for each day. (C) Changes in daily food intake (g/mouse/cage) during the diet. (D) Plasma AST or ALT levels. (E) H&E staining of liver tissues. Scale of 60 μm. (F) Plasma levels of triglycerides, total cholesterol, and glucose. (G) Plasma levels of food intake regulators. (H) *Tm4sf5*, *Apelin*, and *Spp-1/Opn* mRNA levels in liver tissues. See Table 1 for the primers. (I) Immunoblots of liver tissue extracts for the indicated molecules. (J) mRNA analyses from mRNA-Seq data prepared from SNU449-EV (empty vector) and SNU449-TM4SF5 cells. ∗, ∗∗, and ∗∗∗ indicate p ≤ 0.05, p ≤ 0.01, and p ≤ 0.001, respectively. Data are represented as mean ± SD. Data represent three independent experiments. See also Figure S1.

To understand how TM4SF5 causes or initiates NAFLD, we examined whether TM4SF5 expression could influence the food-intake behaviors that might be involved in the earlier stages of the development of TM4SF5-mediated NASH-related features. Male 9-week-old wild-type (WT) and hepatocyte-specific TM4SF5-overexpressing transgenic (*Alb*-TG^Tm4sf5−Flag^) C57BL/6N mice were analyzed for metabolic and pathological parameters during a normal chow diet for 24 h (NCD_1w_) or during the daytime only (9:00 a.m. to 9:00 p.m., daytime feeding, NCD-DF_1w_) for 1 week. We adopted a short diet schedule to examine TM4SF5-mediated effects in the initial stages of NAFLD. Following this period, body weight gain (BWG) was positive in NCD_1w_ and negative in NCD-DF_1w_ independently of genotype (Figure 1A). However, transgenic (TG) mice showed significantly increased food intake during the daytime and reduced food intake during the nighttime compared to WT mice, although food intake per day (g/mouse/day) was comparable between WT and TG mice (Figure 1B). Daily food intake was comparable in the animal groups fed NCD_1w_ but was greater in TG mice fed NCD-DF_1w_ compared to WT mice fed NCD-DF_1w_ (Figure 1C). Interestingly, increased daytime eating in TG mice could be an abnormal food-intake behavior during a generally inactive period for mice. Furthermore, the liver injury parameters AST and ALT were increased in TG mice fed NCD_1w_ but increased insignificantly in TG mice fed NCD-DF_1w_ compared to WT mice (Figure 1D). H&E staining of the liver tissues did not show obvious NAFLD features due to a short diet challenge, but TG mice fed NCD-DF_1w_ showed more damaged hepatocytes compared to WT mice fed NCD-DF_1w_ (Figure 1E, red arrowheads in enlarged image). Among the lipogenic molecule levels we evaluated, sterol regulatory element-binding protein 1 (SREBP1) levels were higher in TG mice fed NCD_1w_ compared to WT mice (Figure S1). Unlike WT and TG mice fed NCD_1w_, which showed comparable plasma triglycerides, total cholesterol, and glucose levels, TG mice fed NCD-DF_1w_ showed significantly higher levels than WT mice fed NCD-DF_1w_ (Figure 1F). Interestingly, WT mice fed NCD-DF_1w_ showed a reduced level of triglycerides (presumably related to less food intake as shown in Figure 1B, right), whereas TG mice were not affected much by NCD-DF_1w_ but had increased blood triglycerides, cholesterol, and glucose levels compared to WT mice fed NCD-DF_1w_ (Figure 1F). Furthermore, hormones that regulate food intake, including leptin, GLP-1, glucagon, ghrelin, and adiponectin, were not significantly different in WT and TG mice fed NCD_1w_, whereas GLP-1 was significantly increased in TG mice fed NCD-DF_1w_ compared to WT mice fed NCD-DF_1w_, and leptin and glucagon were increased in TG mice fed NCD-DF_1w_ compared to TG mice fed NCD_1w_ (Figure 1G). Whereas TG mice fed NCD_1w_ showed significantly increased apelin and decreased SPP-1/OPN levels compared to WT mice fed NCD_1w_, NCD-DF_1w_ caused TG mice to show apelin levels comparable to WT mice but SPP1/OPN levels still lower than WT mice (Figure 1H). This TM4SF5-dependent increase in apelin levels and decrease in SPP1/OPN levels were also shown in immunoblotting of the liver tissue extracts from the associated animal groups (Figure 1I). Furthermore, mRNA sequencing (mRNA-seq) analysis using SNU449 hepatocytes with or without exogenous TM4SF5 expression resulted in higher apelin (*Apln*) and lower Spp-1/Opn (*Spp1*) levels in TM4SF5-positive cells, although the fold changes were not significant (Figure 1J).

**Table 1:** Sequence of primers for qRT-PCR of human or mouse genes

Genes	Forward Primer Sequence (5’ → 3′)	Reverse Primer Sequence (5’ → 3′)
*hTM4SF5*	CTTGCTCAACCGCACTCTAT	ATCCCACACAGTACTATCTCCA
*hSPP1*	TCTGATGAACTGGTCACTGATTT	CTCGGCCATCATATGTGTCTAC
*hAPLN*	GATGGGAATGGGCTGGAAG	AATTTCCTCCGACCTCCCT
*hGDF15*	CTACAATCCCATGGTGCTCAT	TCATATGCAGTGGCAGTCTTT
*hLEPR*	AGATGGTCAACCAGTACAATCC	GGGCTCAGATATGGGATGAATAG
*hADM2*	CGACCTGTGGTCTGGAAG	GGAGAGGCTGACCCATAAC
*hBDNF*	AGTTCGGCCCAATGAAGAA	CCTCCAGCAGAAAGAGAAGAG
*hPEX2*	CATGGTGTATTCCTCTTACTGGTG	GGCCACTCTCCACATAGAGC
*hATGL*	CTCCACCAACATCCACGAG	CCCTGCTTGCACACTCTC
*mApln*	CGAGTTGCAGCATGAATCTGAG	TGTTCCATCTGGAGGCAACATC
*mSpp1*	CTTTCACTCCAATCGTCCCTAC	CAGAAACCTGGAAACTCCTAGAC
*mTm4sf5*	CGAATTGGACCCAAATGCTTAAT	CGCCTCACACAAATTCCAAAG
*mCcl2*	TTAAAAACCTGGATCGGAACCAA	GCATTAGCTTCAGATTTACGGGT
*mCcl5*	CTGCTGCTTTGCCTACCT	TCGAGTGACAAACACGACTG
*mCcl20*	TTGCTTTGGCATGGGTACT	CATACAGACGCCTCTTCCTTC
*mCxcl10*	GGCCATAGGGAAGCTTGAAA	CAGACATCTCTGCTCATCATTCT
*mCxcl1*	GCTGGGATTCACCTCAAGAA	TGGCTATGACTTCGGTTTGG
*mTnf-α*	CCCTCACACTCAGATCATCTTCT	GCTACGACGTGGGCTACAG
*mIl-6*	GAGGATACCACTCCCAACAGACC	AAGTGCATCATCGTTGTTCATACA
*mPpar-α*	AGGCTGTAAGGGCTTCTTTC	GCATTTGTTCCGGTTCTTCTTC
*mAcox1*	GGATGGTAGTCCGGAGAACA	AGTCTGGATCGTTCAGAATCAAG
*mAcadsb*	TGCTCCTCTGGTTTCCTCTA	GTCCCTCCATATTGTGCTTCA
*mAcadl*	CTCAGGACACAGCAGAACTATT	GCTCTTGCATGAGGTAGTAGAA
*mCpt1a*	CCTGGGCATGATTGCAAAG	ACGCCACTCACGATGTTCTTC
*mCpt2*	TCTTCCTGAACTGGCTGTCA	GTACCCACCATGCACTACCA
*mGcgr*	GAACCTGTTTGCGTCCTTTG	CTGAGGTCATCGCCAATCTT
*mHmgcr*	CCAGAAGCTTTCGTCAGTAGAG	CTCTGCTTGTAGTCTCTGCTTC
*mSrebp2*	GGTACGCTGGTTACTCAAGAAG	GCTCTTAGCCTCATCCTCAAAG
*mPex2*	AAAAATGATTCTTCTCTCAACCTGA	TGCACACAGCATACCACAGTT
*mAtgl*	TGACCATCTGCCTTCCAGA	TGTAGGTGGCGCAAGACA

### TM4SF5-dependent apelin expression is linked to the regulation of SPP-1/OPN expression via AKT1/ELK1 activity

**Figure 2:**
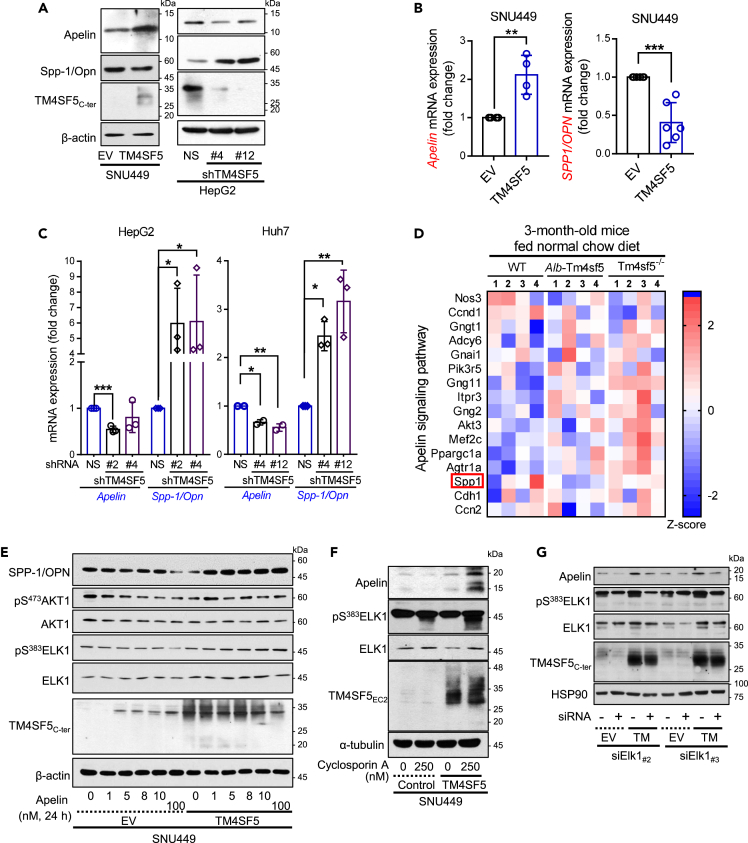
TM4SF5-dependent apelin expression is linked to the regulation of SPP-1/OPN expression via AKT1/ELK1 activity (A–C) SNU449 hepatocytes transfected with empty vector (EV), TM4SF5 (A, B), or HepG2 (A, C) or Huh7 (C) cells transfected with shRNA against non-specific (NS) or the TM4SF5 sequence (#2 or #4, see Table 2) were harvested, prior to immunoblots (A) or qRT-PCR (B, C) for the indicated molecules. ∗, ∗∗, and ∗∗∗ indicate p ≤ 0.05, p ≤ 0.01, and p ≤ 0.001, respectively. Data are represented as mean ± SD. (D) Heatmap for molecules related to the apelin signaling pathway from the mRNA-Seq analysis using liver tissues of 3-month-old male WT, *Alb*-TG^Tm4sf5^, and *Tm4sf5*^−/−^ KO mice fed normal chow *ad libitum*. (E) SNU449 cells as in (A) were treated with apelin at different concentrations for 24 h before whole cell lysate preparation and immunoblots for the indicated molecules. (F) Cyclosporin A were treated to SNU449 cells (0 or 250 nM for 24 h), before whole cell harvests and immunoblots. (G) SNU449 cells stably expressing empty vector (EV) or TM4SF5 expression vector were transiently transfected with siRNA against control sequence (−) or ELK1 sequences (#2 or #3, see Table 2), before harvests and immunoblots. Data shown represent three independent experiments. See also Figure S2.

We next examined whether TM4SF5 transfection into hepatocytes affected apelin and SPP-1/OPN expression levels. Transient transfection of TM4SF5 into SNU449 hepatocytes increased apelin levels and decreased SPP-1/OPN peptide levels (Figure 2A), parallel with their mRNA levels (Figure 2B), indicating that the TM4SF5-mediated effects on both molecules might occur at the transcriptional level. Furthermore, suppression of endogenous TM4SF5 in HepG2 and Huh7 hepatocytes reversed the TM4SF5-mediated effects (Figure 2C). Using these SNU449, HepG2, and Huh7 hepatocytes with or without TM4SF5 expression (i.e., overexpression or suppression systems), we commonly observed TM4SF5-promoted *Apln* mRNA levels and TM4SF5-reduced *Spp1* levels, whereas the mRNA levels of other hormone molecules like GDF15, leptin receptor (LEPR), adrenomedullin 2 (ADM2), or BDNF were not significantly affected (Figure S2). When we examined the apelin-linked genes in the mRNA-seq datasets from 3-month-old WT, TG, and KO mice, we found that the level of *Spp1* negatively correlated with *Tm4sf5* expression (Figure 2D). Furthermore, when we treated hepatocyte SNU449 (empty vector [EV] as a control) or SNU449-TM4SF5 cells with apelin, SPP-1/OPN levels in SNU449-EV cells were gradually reduced in an apelin dose-dependent manner, whereas SNU449-TM4SF5 cells showed increased SPP-1/OPN levels that eventually became higher than untreated or apelin-treated SNU449-EV cells (Figure 2E). Such changes in SPP-1/OPN expression upon apelin treatment were well correlated with the phospho-S^473^ in AKT1 (also known as protein kinase B, pS^473^AKT1) and phospho-S^383^ELK1 (pS^383^ELK1 transcription factor) changes (Figure 2E), thereby suggesting that TM4SF5 can control the expression of apelin and SPP-1/OPN via regulation of AKT1 and ELK1 activities in hepatocytes. Activation of ELK1 by cyclosporin A^25^ increased TM4SF5-mediated apelin expression only in TM4SF5-positive cells but not in TM4SF5-negative cells (Figure 2F). Furthermore, suppression of ELK1 abolished TM4SF5-promoted apelin expression (Figure 2G).

**Table 2:** shRNA targeting sequences against *TM4SF5*

siRNA or shRNA targeting sequence against human *TM4SF5* or *ELK1*	Sequence (5’→ 3′)
shRNA-NS	CCTAAGGTTAAGTCGCCCTCGCTCGAGCG AGGGCGACTTAACCTTAGG
shTM4SF5 #2	CCGGACCATGTGTACGGGAAAATGTGCCT CGAGGCACATTTTCCCGTACACATGGTTTTTTG
shTM4SF5 #4	CCGGCCATCTCAGCTTGCAAGTCCTCGAGG ACTTGCAAGCTGAGATGGTTTTTG
shTM4SF5 #12	CCGGTGGACCCAGATGCTTAATGAACTCGAG TTCATTAAGCATCTGGGTCCATTTTTG
siELK1 #2	CGUAAUUCAUGUUGGUCUUGUUCUUGC
siELK1 #3	GAACUGAAAAUUCAUGUUUGGUAUCAA

### *Alb*-TG^Tm4sf5−Flag^ mice fed HFD showed greater food intake than KO mice

**Figure 3:**
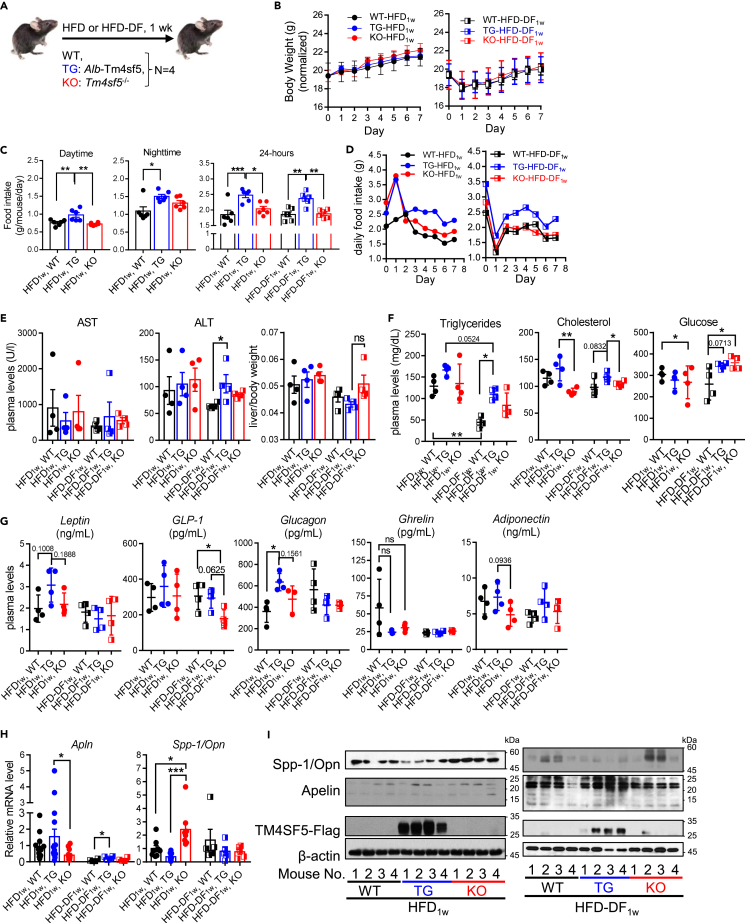
*Alb*-TG^Tm4sf5-Flag^ mice fed HFD showed greater food intake than KO mice Male WT, *Alb*-TG^Tm4sf5−Falg^, and *Tm4sf5*^−/−^ KO mice (n = 4) were fed high-fat diet (60% kcal fat, HFD_1w_) *ad libitum* during an entire day (i.e., 24 h) or daytime only (i.e., 9:00 a.m. to 9:00 p.m., HFD-DF_1w_) for 1 week. (A) Scheme for the experimental protocol. (B) Normalized body weights (g). (C) Food intake (g/mouse/day) for either daytime only, nighttime only, or an entire day (24 h) of animals fed HFD_1w_. (D) Daily food intake (g) of animals fed HFD_1w_ or HFD-DF_1w_. (E) Plasma AST or ALT levels and liver/body weight ratio of the animals. (F) Plasma triglycerides, cholesterol, and glucose levels of the animals. (G and H) Levels of food intake regulators including apelin and SPP-1/OPN measured in animal plasma samples. ∗, ∗∗, and ∗∗∗ indicate p ≤ 0.05, p ≤ 0.01, and p ≤ 0.001, respectively. ns indicates non-significance. Data are represented as mean ± SD. (I) Whole tissue extracts of livers from the animals fed HFD_1w_ or HFD-DF_1w_ were processed for immunoblots for the indicated molecules. Two pieces of a liver from an animal were processed for the analyses. The data shown represent three isolated experiments.

We next examined how *ad libitum* HFD for an entire day (HFD_1w_) or a daytime-feeding-only pattern (HFD-DF_1w_) for 1 week could affect the animals (n = 4, Figure 3A). Again, the HFD challenge was shortly performed to understand how TM4SF5 expression affected liver damage as an earlier pathological feature of NAFLD. Although normalized BWG was not different between male WT, TG, or KO mice fed HFD_1w_ or HFD-DF_1w_ (Figure 3B), changes in food intake (g/mouse/day) were different. TG mice showed greater food intake during either daytime or nighttime than WT or KO mice under any diet patterns (Figure 3C). Furthermore, daily food intake during the experimental period was greater in TG mice fed HFD_1w_ or HFD-DF_1w_ compared to WT or KO mice fed the same diets (Figure 3D). The same observation with HFD_1w_ confirmed that TG mice had abnormally greater food intake during the daytime. AST and ALT levels and liver weights were not different between the animals fed HFD_1w_, whereas ALT levels were higher in TG mice fed HFD-DF_1w_ compared to WT and KO mice (Figure 3E). Because a short diet challenge of about 1 week may not lead to enough liver inflammation to enhance TM4SF5 expression in WT mice,^21^^,^^24^ the phenotypes of WT mice might be similar to those of KO mice. In addition, the mean values of plasma triglycerides and the total cholesterol levels of TG mice were higher than those of WT or KO mice fed either HFD_1w_ or HFD-DF_1w_, although the levels of animals fed HFD-DF_1w_ were lower than those of animals fed HFD_1w_ (Figure 3F). KO mice fed HFD_1w_ showed lower mean blood glucose levels compared to WT mice, whereas WT mice fed HFD-DF_1w_ showed lower levels than TG and KO mice, indicating no clear TM4SF5 dependency of blood glucose levels in animals fed HFD-DF_1w_ (Figure 3F, right). While orexigenic glucagon levels in TG mice fed HFD_1w_ were higher than those in WT or KO mice fed the same diet, other hormones (leptin, GLP-1, ghrelin, and adiponectin) were not significantly different between animal groups (Figure 3G). In addition, anorexigenic GLP-1 levels in WT and TG mice were higher than those in KO mice under the HFD-DF_1w_ condition (Figure 3G). The hepatic *Apln* mRNA levels of TG mice fed HFD_1w_ were significantly higher than those in KO mice, whereas *Spp1* mRNA levels in KO mice were significantly higher than those in TG and WT mice (Figure 3H), consistent with the observations in animals fed NCD_1w_ and in cell lines (Figures 1 and 2). In addition, HFD-DF_1w_ decreased *Apln* mRNA levels in all animal groups, although TG mice showed significantly higher levels compared to WT mice (Figure 3H, left). In immunoblotting, liver tissue extracts showed lower SPP-1/OPN levels and higher apelin levels in TG mice fed HFD_1w_ or HFD-DF_1w_ than in KO mice (Figure 3I). Thus, TM4SF5 expression in hepatocytes appeared to cause greater food intake during the daytime, and this greater food intake was correlated with increased apelin and decreased SPP-1/OPN.

### *Alb*-TG^Tm4sf5−Flag^ mice fed *ad libitum* HFD_1w_ showed inflammatory livers

**Figure 4:**
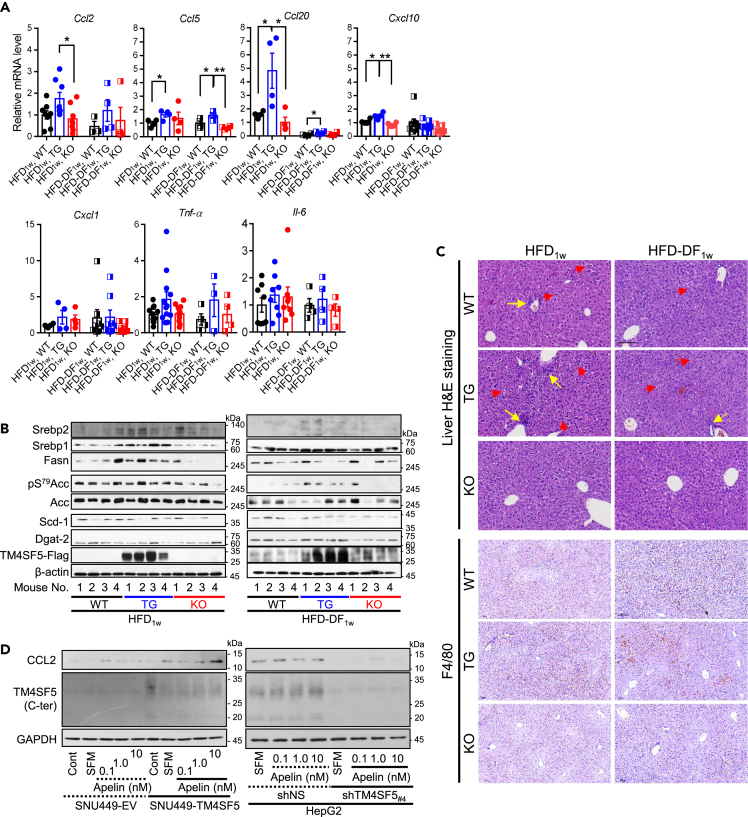
*Alb*-TG^Tm4sf5−Flag^ mice fed HFD *ad libitum* showed inflammatory livers (A and B) Male WT, *Alb*-TG^Tm4sf5−Falg^, and *Tm4sf5*^−/−^ KO mice (n = 4) were fed HFD_1w_*ad libitum* during an entire day (i.e., 24 h) or daytime only (i.e., 9:00 a.m. to 9:00 p.m., HFD-DF_1w_) for 1 week before various analyses. Liver tissues were processed for qRT-PCR to measure the relative mRNA levels for the indicated molecules (A), immunoblots (B), or H&E staining and immunohistochemistry using anti-F4/80 antibody. (C) Scale of 100 μm. (D) Subconfluent TM4SF5-negative SNU449-EV or HepG2-shTM4SF5_#4_ and TM4SF5-positive SNU449-TM4SF5 or HepG2-shNS were harvested for immunoblots for the indicated molecules. ∗ and ∗∗ depict p ≤ 0.05 and p ≤ 0.01, respectively. Data are represented as mean ± SD. Yellow arrows indicate infiltrated immune cells, and red arrowheads indicate damaged hepatocytes. Data shown represent three isolated experiments.

We next examined the inflammation in the livers of animals fed HFD_1w_ or HFD-DF_1w_. The mRNA levels of chemokine ligand 2 (*Ccl2*), *Ccl20*, *Ccl5,* and chemokine (C-X-C motif) ligand 10 (*Cxcl10*) significantly increased in the livers of TG mice fed HFD_1w_ compared to WT or KO mice, whereas the mRNA levels of *Cxcl1*, tumor necrosis factor alpha (*Tnf-α*), and interleukin 6 (*Il6*) were comparable among animal groups (Figure 4A). In all groups, animals fed HFD-DF_1w_ had decreased levels compared to animals fed HFD_1w_, although TG mice fed HFD-DF_1w_ still had higher levels than KO mice (Figure 4A). These observations suggest that greater food intake by TG mice could lead to an inflammatory environment in the liver. In addition, immunoblotting of the liver tissue extracts showed enhanced expression of SREBP1, SREBP2, and fatty acid synthase (FASN) in WT and TG mice compared to KO mice, and KO mice showed better bioenergetic status as indicated by a lower pS^79^Acc level compared to WT or TG mice (Figure 4B), indicating that TM4SF5-dependent lipogenesis was favored in WT and TG mice. We further examined the liver tissues with H&E and immunohistochemistry. H&E staining showed more hepatocyte damage (red arrowheads) and immune cell infiltration (yellow arrows) in the livers of TG mice fed HFD_1w_ compared to WT or KO mice, although hepatocyte damage was slightly reduced in the animals fed HFD-DF_1w_ (Figure 4C, upper panels), indicating that time-restricted feeding (DF) might play a role in slightly reducing the *ad libitum* HFD-mediated effects on TG mice, as also shown in cytokine/chemokine and lipogenic molecule levels (Figures 4A and 4B). In addition, immunohistochemical staining of F4/80 to indicate murine macrophages showed stronger stains in the TG mice fed HFD_1w_ or HFD-DF_1w_ compared to KO mice (Figure 4C, lower panels). Furthermore, apelin treatment of TM4SF5-positive SNU449-TM4SF5 or HepG2 cells led to the induction of CCL2, unlike TM4SF5-negative or suppressed counterparts (Figure 4D), indicating that apelin-mediated proinflammation is possible in TM4SF5-positive hepatocytes. Therefore, hepatic TM4SF5 might be involved in the earlier stages of NASH feature development via HFD_1w_, including enhanced blood lipid, inflammation, and SREBP1/FASN levels caused by abnormal daytime food intake and apelin expression.

### *Alb*-TG^Tm4sf5−Flag^ mice fed HFD_1w_ have peroxisomal β-oxidation and mTOR activation

**Figure 5:**
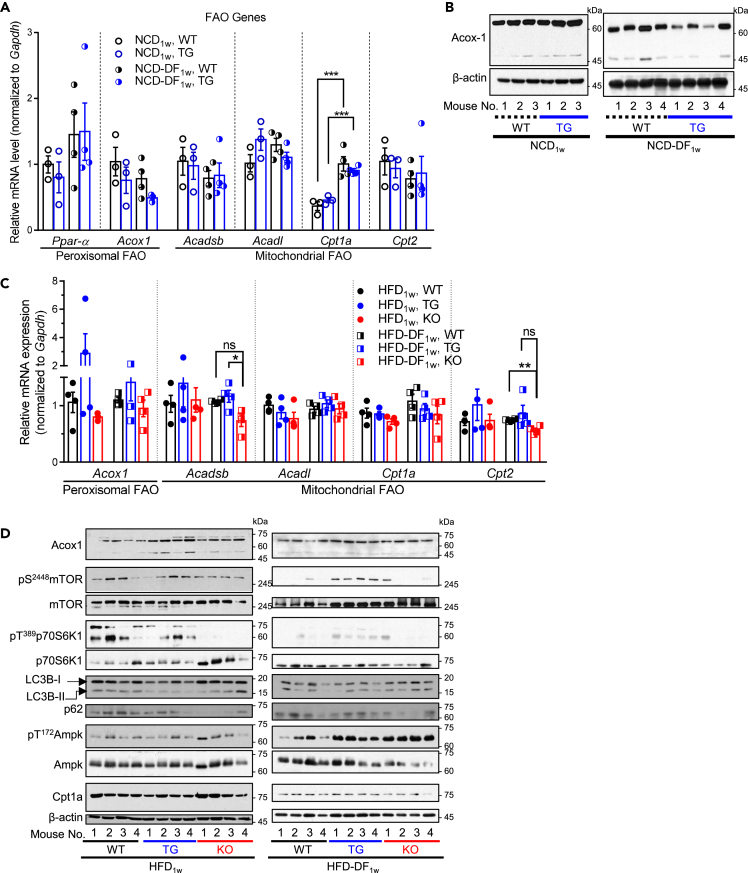
*Alb*-TG^Tm4sf5−Falg^ mice fed HFD_1w_ showed peroxisomal β-oxidation and mTOR activation (A–D) Male WT, *Alb*-TG^Tm4sf5−Flag^, and *Tm4sf5*^−/−^ KO mice (n = 4) were fed NCD_1w_ or HFD_1w_*ad libitum* for an entire day (i.e., 24 h) or daytime only (i.e., 9:00 a.m. to 9:00 p.m.), NCD-DF_1w_, or HFD-DF_1w_ before analyses. Liver tissues of the animals were processed for qRT-PCR (A, C) or immunoblots (B, D) for the indicated FAO-related molecules. ∗, ∗∗, and ∗∗∗ depict p ≤ 0.05, p ≤ 0.01, and p ≤ 0.001, respectively. ns indicates non-significance. Data are represented as mean ± SD. Data shown represent three independent experiments.

Time-restricted feeding or HFD leads to a fatty liver via an increase in acetyl-coenzyme A (CoA) levels by peroxisomal β-oxidation, mTOR activation, and autophagy and lipophagy inhibition.^26^ Because we observed greater hepatocyte damage with inflammation and lipogenic enzyme activation in the livers of TG mice fed HFD_1w_, we wondered whether the TM4SF5-mediated effects involved peroxisomal β-oxidation. Therefore, we analyzed the levels of molecules involved in β-oxidation (fatty acid oxidation [FAO]) through peroxisomes or mitochondria. The hepatic mRNA levels of peroxisome proliferator-activated receptor alpha (*Ppar-a*) or peroxisomal acyl-CoA oxidase 1 (*Acox1*) for peroxisomal FAO and *Acadsb*, acyl-CoA dehydrogenase long chain (*Acadl*), carnitine palmitoyltransferase 1a (*Cpt1a*), or *Cpt2* for mitochondrial FAO in TG mice fed NCD_1w_ were comparable to those in WT mice, although NCD-DF_1w_ caused increased *Ppar-a* and *Cpt1a* levels independent of genotype (Figure 5A), indicating that FAO was favored by the time-restricted diet of NCD-DF_1w_ independently of TM4SF5. However, in liver tissue immunoblots, ACOX1, the first enzyme involved in peroxisomal FAO, was comparable in WT and TG mice fed NCD_1w_ but was lower in the livers of TG mice fed NCD-DF_1w_ compared to WT mice (Figure 5B). When mRNA levels were analyzed using the liver tissues of mice fed HFD_1w_ or HFD-DF_1w_, mean values of *Acox1*, *Acadsb*, and *Cpt2* mRNA levels were slightly higher in TG mice compared to WT or KO mice (Figure 5C). Furthermore, liver tissue immunoblotting showed higher ACOX1 levels in TG mice than in WT or KO mice fed HFD_1w_ or HFD-DF_1w_. In contrast, Cpt1a levels were not higher in TG mice livers; instead, they were less than or comparable to those in WT or KO mice (Figure 5D). In addition, TG mice fed HFD_1w_ or HFD-DF_1w_ showed higher mTOR phosphorylation and lower autophagic LC3B-II levels compared to WT or KO mice, whereas AMPK phosphorylation levels were lower in TG mice compared to KO mice (Figure 5D). These observations suggest that TG mice fed HFD_1w_ have an increased tendency for peroxisomal FAO preference, mTOR activation, and autophagy inhibition compared to WT and KO mice.

### Apelin treatment of *Alb*-TG^Tm4sf5−Flag^ mice fed HFD_2w_ could promote NAFLD features

**Figure 6:**
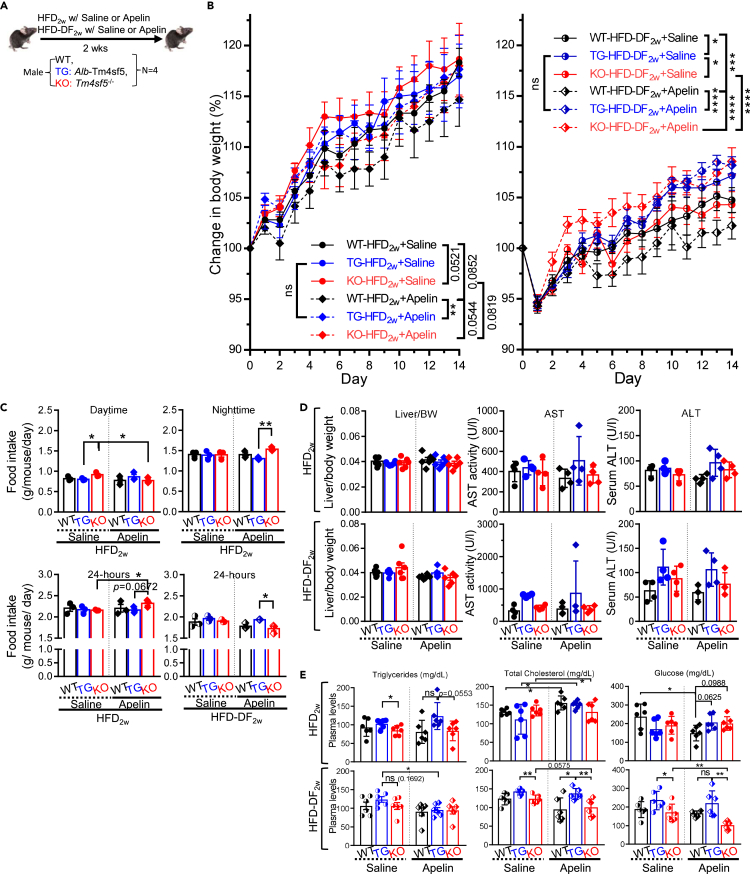
Apelin treatment of *Alb*-TG^Tm4sf5−Flag^ mice fed HFD_2w_ promoted NAFLD features Male WT, *Alb*-TG^Tm4sf5−Falg^, and *Tm4sf5*^−/−^ KO mice (n = 4) were fed HFD_2w_*ad libitum* during an entire day (i.e., 24 h) or daytime only (i.e., 9:00 a.m. to 9:00 p.m., HFD-DF_2w_) for 2 weeks with saline or apelin treatment (300 μg/injection/kg/day) via intraperitoneal injection before analyses. (A) Scheme of the experimental protocol. (B) Changes in body weights (%, body weight gains; BWG) of the animals. (C) Food intake (g/mouse/day) for either daytime only, nighttime only, or an entire day (24 h) of animals fed HFD_2w_ or HFD-DF_2w_. (D) Plasma AST or ALT levels and liver/body weight ratio of the animals. (E) Levels of triglycerides, cholesterol, and glucose levels in the animal plasma samples. ∗, ∗∗, and ∗∗∗ indicate p ≤ 0.05, p ≤ 0.01, and p ≤ 0.001, respectively. ns indicates non-significance. Data are represented as mean ± SD. The data shown represent three isolated experiments. See also Figure S3.

Next, we wondered if apelin treatment of animals fed HFD for a longer period (i.e., 2 weeks, HFD_2w_) could also affect the food-intake behaviors and more advanced pathological status in the liver (Figure 6A). Apelin (300 μg/kg/day, [Pyr^1^]-Apelin-13) was administered via intraperitoneal injection at 9:00 a.m. every day. With HFD_2w_(+Saline), all animal groups showed comparable BWG, but KO mice showed BWG insignificantly greater than WT mice (p = 0.0521). In contrast, with HFD-DF_2w_(+Saline), TG mice showed significantly greater BWG than WT and KO mice, with greater differences in BWG as time passed after 1 week (Figure 6B). Interestingly, exogenous apelin treatment in addition to HFD_2w_ did not change BWG in TG mice but decreased BWG in WT (p = 0.0852) and KO (p = 0.0819) mice while keeping BWG for KO mice higher than that of WT mice (p = 0.0544; Figure 6B, left). Whereas HFD-DF_2w_+Saline led to TG mice with significantly greater BWG than WT or KO mice, HFD-DF_2w_+Apelin caused TG and KO mice to show comparable BWG that was significantly greater than that of WT mice (Figure 6B, right). Interestingly, compared to HFD-DF_2w_+Saline, HFD-DF_2w_+Apelin led to a significant increase in the BWG of KO mice, no significant change in the BWG of TG mice, and a significant decrease in the BWG of WT mice (Figure 6B, right). Furthermore, daytime-only feeding (DF) reduced the BWG in all animal groups compared to mice fed HFD_2W_ or HFD_2W_+Apelin (Figure 6B). These observations indicate that apelin treatment of animals fed HFD_2W_ might cause less BWG in WT and KO mice but did not alter BWG in TG mice, whereas apelin treatment of animals fed HFD-DF_2w_ might cause TM4SF5-independently increased BWG in KO mice, although BWG was not altered in TG mice and was decreased in WT mice compared to saline-treated animals. Thus, TG mice showed highly maintained BWG independent of apelin treatment in addition to HFD_2w_ or HFD-DF_2w_.

Consistent with decreased BWG in KO mice fed HFD_2w_ upon apelin treatment, KO mice showed higher daily food intake during the daytime than TG mice, which was abolished (rather than lowered) upon apelin treatment. In contrast, apelin treatment could cause significantly increased food intake during the nighttime to a level higher than TG mice (Figure 6C, upper), leading to higher overall food intake per day in KO mice than in TG mice (p = 0.0672; Figure 6C, lower left). Thus, HFD_2w_+Apelin had a TM4SF5-independent anorexigenic effect in KO mice. Consistently, KO mice fed HFD-DF_2w_+Saline had decreased food intake to a level significantly lower than that of TG mice (Figure 6C, lower right). Therefore, HFD-DF_2w_ could reduce the food intake (for an entire day) in all animal groups compared with animals fed HFD_2W_, but this influence was greater in TM4SF5-negative KO mice. In addition, the anorexigenic effects of exogenous apelin during the daytime and the orexigenic effects during the nighttime appeared obvious in KO mice fed HFD_2w_ or HFD-DF_2w_, presumably because TG or WT mice might have endogenous apelin expression, so exogenous apelin might not be additional (Figure 6C). On the other hand, liver/body weight ratios were not different among the animals during HFD_2w_ with saline or apelin treatment (Figure 6D, left). Furthermore, the AST or ALT levels of TG mice fed HFD_2w_ or HFD-DF_2w_ with saline or apelin treatment showed increased trends (though not significant) compared to WT or KO mice (Figure 6D, middle and right). When plasma triglyceride, cholesterol, and glucose levels were measured, TG mice fed HFD_2w_ or HFD-DF_2w_ with or without apelin treatment showed higher levels compared to WT or KO mice (Figure 6E). Whereas TG mice fed HFD_2w_ or HFD-DF_2w_ showed increased or slightly decreased/maintained levels upon apelin treatment, respectively, KO mice showed maintained or decreased levels (Figure 6E). Furthermore, time-restricted feeding (i.e., DF) caused TG mice to have higher levels (in mg/dl) but reduced the levels in WT and KO mice (Figure 6E). Thus, DF led to less food intake (Figure 6C), which caused KO mice to show lower plasma cholesterol and glucose levels (even more obviously upon apelin treatment) but did not affect plasma cholesterol and glucose levels in TG mice (Figure 6E).

### DF or apelin treatment could more strongly influence TG mice toward NAFLD features

**Figure 7:**
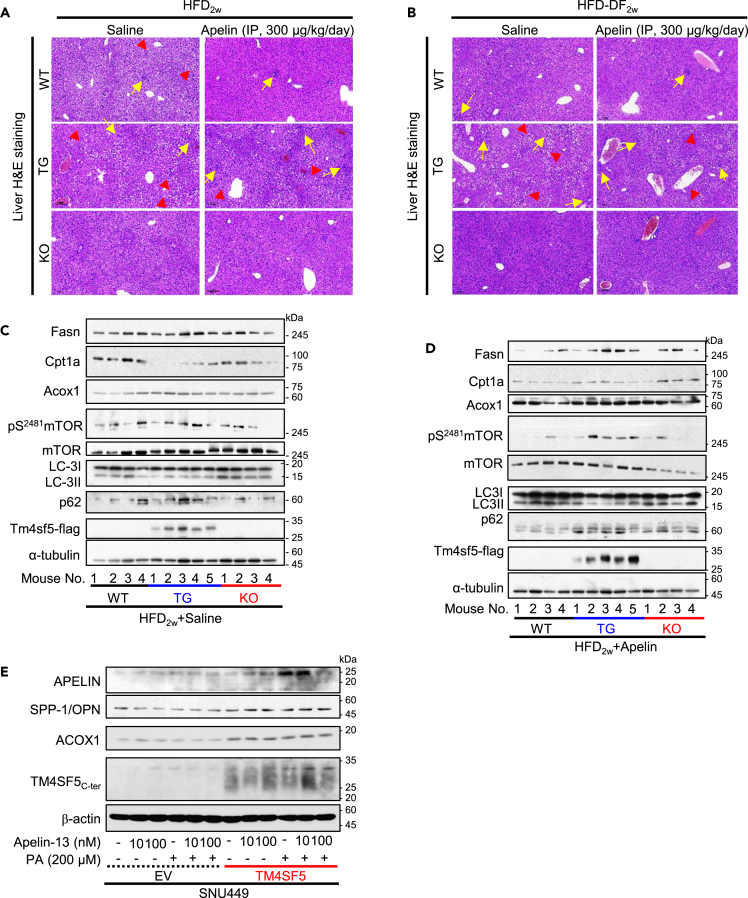
Daytime feeding or apelin treatment could influence TG mice for NAFLD features (A–D) Liver tissues of mice fed HFD_2w_+Saline (A, C) or HFD_2w_+Apelin (B, D) were processed for H&E staining, and representative images were shown (A, B), or for whole tissue extracts before immunoblots for the indicated molecules (C, D). Yellow arrows indicate infiltrated immune cells, and red arrowheads indicate damaged hepatocytes. Note the major fat droplet accumulation in TG mice and minor accumulation in WT mice. Scale of 100 μm. (E) SNU449-EV and SNU449-TM4SF5 cells were treated with apelin and/or palmitic acid for 24 h before harvests and immunoblots. The data shown represent three isolated experiments.

Apelin-mediated effects in TG mice fed HFD_2w_ (i.e., HFD_2w_+Apelin) were also observed in H&E staining and immunoblots of the livers. TG mice fed HFD_2w_ with or without apelin treatment showed hepatic fat droplet depositions, hepatocyte damages, and slight immune cell infiltrations, whereas KO mice fed HFD_2w_ with or without apelin treatment showed rare or no fat droplets (Figure 7A). With HFD-DF_2w_, TG mice with or without apelin treatment had fat droplet depositions, immune cell infiltration (yellow arrows), and hepatocyte damages (red arrowheads), whereas WT mice showed slight fat accumulation and inflammation and KO mice did not (Figure 7B). Although ACOX1 levels in TG mice were not different from those in KO mice during HFD_2w_+Saline, TG mice showed slightly higher FASN levels and lower LC3B-II levels compared to KO mice (Figure 7C). In addition, TG mice fed HDF_2w_+Apelin showed higher FASN and lower Cpt1a levels compared to KO mice. Furthermore, TG mice concomitantly showed higher levels of ACOX1, mTOR phosphorylation (pS^2481^mTOR), and p62 expression and lower levels of Unc-51 Like Autophagy Activating Kinase 1 (ULK1) phosphorylation (pS^555^Ulk1) and LC3B-II compared to KO mice (Figure 7D), suggesting that lipogenesis, mTOR activation, and concomitant autophagy inhibition are preferred in TG mice. Meanwhile, the TM4SF5-dependent ACOX1 levels were not correlated with Peroxisomal Biogenesis Factor 2 (PEX2) and lipases adipose triglyceride lipase (ATGL) levels (Figure S3) for a decrease in lipolysis,^27^ suggesting that TM4SF5-mediated ACOX1 might not be linked to lipolysis activity. Furthermore, treatment of TM4SF5-null SNU449-EV cells with apelin alone or together with palmitic acid did not have obvious changes in apelin, SPP-1/OPN, or ACOX1, whereas treatment of SNU449-TM4SF5 cells showed increased apelin, decreased SPP-1/OPN, and higher ACOX1 levels (Figure 7E). These observations suggest that HDF_2w_, HFD-DF_2w_, and/or exogenous apelin treatment could cause TM4SF5-positive hepatocytes and mice to be steatotic and even steatohepatitic, whereas the same paradigms applied to TM4SF5-negative hepatocytes or mice could not.

### Human NAFLD patients increased apelin as NAFLD developed

**Figure 8:**
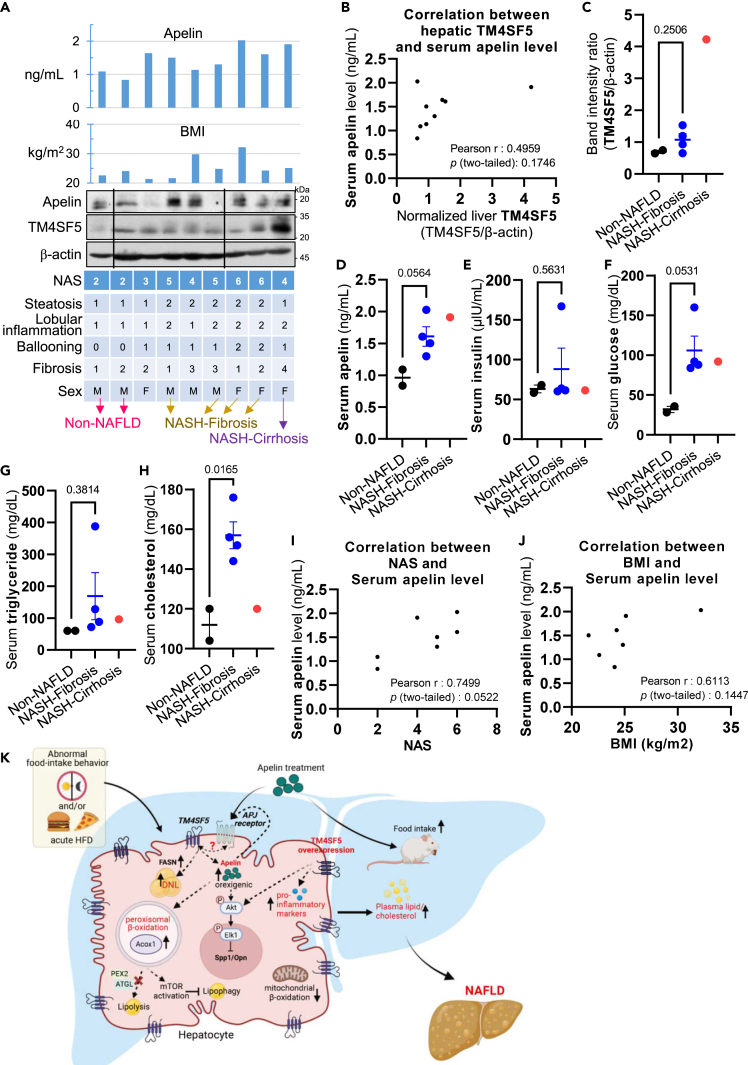
Human NAFLD patients increased apelin as NAFLD developed (A) TM4SF5-related molecules and pathological features in 9 sample pairs of liver tissue and serum from non-NAFLD, NASH-fibrosis, or NASH-Cirrhosis patients. (B) Correlation between TM4SF5 expression (band intensity normalized to β-actin band intensity) in liver tissues and serum apelin levels in the 9 sample pairs (p = 0.1746). (C–H) Correlation between TM4SF5 expression (band intensity normalized to β-actin band intensity) in liver tissues (C), serum apelin (D), insulin (E), glucose (F), triglycerides (G), and total cholesterol (H) levels in the 7 sample pairs (2 non-NAFLD, 4 NASH-Fibrosis, and 1 NASH-Cirrhosis sample pairs). (I and J) Correlation between serum apelin levels and NAS (E) or BMI (F) in the 7 sample pairs (p = 0.0522 or 0.1447, respectively). The unpaired two-tailed Student’s *t* test was performed. Data are represented as mean ± SD. (K) Working model for hepatocyte TM4SF5-mediated abnormal food intake and apelin expression, leading to DNL, a pro-inflammatory environment, and peroxisomal β-oxidation, which inhibit lipophagy during the initial stages of NAFLD development. See also Figure S4.

We further confirmed the relationships between TM4SF5-related molecules and features in different hepatic pathological stages using paired patient liver and serum samples. Out of 12 sample pairs, 9 sample pairs that had been clinically classified with NAFLD stages depending on NAFLD activity score (NAS) with BMI were analyzed for immunoblots and serum apelin levels (Figure 8A). Among the 9 sample pairs, TM4SF5 expression (as determined by band intensity normalized to β-actin band intensity) tended to increase as serum apelin levels increased, although the difference between groups was not statistically significant (p = 0.1746, Figure 8B). We next examined how TM4SF5 expression could differ depending on the liver disease status. After removing two samples that were NAS 3 and 4 for obscure NASH phenotypes, the remaining 7 sample pairs (2 non-NAFLD, 4 NASH-Fibrosis, and 1 NASH-Cirrhosis) showed increased hepatic TM4SF5 expression and serum apelin levels as liver disease became severe, although the difference between groups were not statistically significant (p = 0.2506 and p = 0.0564, respectively; Figures 8C and 8D). A similar increase in TM4SF5 and apelin levels during obesity or NAFLD development was also found in a public dataset of GSE48325 (Figure S4A). In addition, serum insulin and glucose levels were unchanged (p = 0.5631) and increased non-significantly (p = 0.0531), respectively, in NAFLD patients compared to non-NAFLD individuals, indicating insulin resistance in NAFLD patients (Figures 8E and 8F). Furthermore, although serum triglyceride levels were not changed in NAFLD-Fibrosis patients (p = 0.3814; Figure 8G), total cholesterol levels were increased compared to non-NAFLD individuals (p = 0.0165; Figure 8H). Serum apelin levels were also correlated with NAS (p = 0.0522; Figure 8I) and body mass index (BMI) though not significantly (p = 0.1447; Figure 8J). In addition, TM4SF5 expression was not associated with NAS, serum insulin, or BMI alone (Figures S4B–S4D). Furthermore, serum insulin levels were not associated with NAS or BMI alone (Figures S4E and S4F). Serum insulin levels were negatively correlated with TM4SF5 expression and BMI, although the changes were not statistically significant (Figure S4). In summary, serum apelin level appeared to be positively correlated with hepatic TM4SF5 level (p = 0.1746, Figure 8B) or NAS (p = 0.0522; Figure 8I), although the results were not statistically significant because of the small sample size.

## Discussion

This study reveals that TM4SF5 expression in hepatocytes can promote metabolically pathological features in the liver via abnormal food-intake behaviors, increased apelin for an orexigenic role, and changes in SPP-1/OPN levels, even during short NCD or HFD periods of 1 or 2 weeks. Time-restricted DF during HFD_1w_ or HFD_2w_ also resulted in aberrantly higher blood lipid and glucose levels in hepatocyte-specific TM4SF5-overexpressing TG mice compared to WT and KO mice. Exogenous apelin treatment also resulted in orexigenic effects, especially in KO mice during the nighttime but had anorexigenic effects during the daytime, resulting in a TM4SF5-independent orexigenic effect on food intake for an entire day. In contrast, the treatment of TG mice did not cause significant changes, possibly due to saturated endogenous apelin effects. Meanwhile, apelin treatment during HFD-DF_2w_ caused KO mice to have less food intake and lower blood cholesterol and glucose levels but did not influence TG mice. Such obvious TM4SF5-dependent features that maintained greater BWG and food intake under NCD, HFD, DF, and/or apelin treatment conditions were also correlated with higher enzyme levels for *de novo* lipogenesis, inflammatory chemokine mRNAs, hepatocyte damages, and lipid droplet depositions in livers compared to those in WT or KO mice. These steatotic or earlier steatohepatitic features in TG mice via abnormal daytime food intake and apelin induction appeared correlated with not only enhanced peroxisomal β-oxidation, mTOR activation, and autophagy inhibition but also more lipogenesis, inflammation, and blood lipid levels, even following HFD for 2 weeks. Accordingly, peroxisomal β-oxidation is a major source of acetyl-CoA that regulates the mTOR-autophagy axis, leading to the promotion of hepatic steatosis.^26^ In addition, analyses using liver and serum samples from non-NAFLD, NAFLD-NASH, and NASH-Cirrhosis patients revealed that TM4SF5 tended to increase as NAFLD developed along with serum apelin level enhancement, insulin resistance, and cholesterol enrichment. Thus, hepatic TM4SF5 overexpression may be involved in triggering earlier NAFLD features even following short HFD challenges (Figure 8K).

Here we observed that apelin appeared orexigenic in hepatocyte-specific TM4SF5-overexpressing mice. Apelin and its receptor angiotensin II receptor like-1 (APJ receptor) appear to be involved in liver diseases including fibrosis and cirrhosis.^28^ Although potential roles for apelin in homeostasis and energy metabolism have been suggested,^12^ apelin produced in adipocytes and hepatocytes has had inconsistent orexigenic or anorexigenic roles. Serum apelin level is increased in obesity and insulin-resistant status,^13^ whereas apelin deficiency increases adiposity and blood fatty acid levels,^14^ and apelin overexpression is resistant to obesity.^15^ Such inconsistent apelin effects on food intake might be explained by differences in TM4SF5 expression in the experimental conditions. Although we did not measure the apelin production in TG mice separately during the daytime and nighttime, TG mice fed NCD_1w_ or HFD_1w_ showed abnormally greater food intake during the daytime, an inactive period for mice, which could be linked to higher AST and ALT values. In addition, under conditions without TM4SF5 expression, apelin appeared orexigenic during the nighttime but anorexigenic during the daytime, as shown in KO mice fed HFD_2w_. During HFD_2w_, additional exogenous apelin treatment of KO mice caused less food intake during the daytime but more during the nighttime, leading to greater food intake for an entire day. In both HFD_2w_ and HFD-DF_2w_, apelin treatment led TG mice to show more daytime eating than WT or KO mice, further supporting an apelin influence on TM4SF5-promoted abnormal food intake during the daytime. The effects of DF specifically on food intake (via restricting the food available only during the daytime), blood lipid, and glucose levels appeared more obvious in HFD_2w_ than in HFD_1w_. Therefore, the effects of either DF or apelin treatment on triggering NAFLD features could be more relevant in TG mice than *Tm4sf5*^*−/−*^ KO mice, supporting TM4SF5-mediated or dependent effects. As the timing of food intake can be important for abnormal metabolism-related pathologies including steatosis, steatohepatitis, and obesity,^6^ TM4SF5-mediated increased food intake during the daytime may be involved in the initiation or promotion of NAFLD-associated pathological features including hepatic fat deposition, hepatocyte damage, and inflammation.^21^ Consistently, analyses of liver tissue and serum samples from healthy or NAFLD patients revealed that serum apelin levels were correlated (though not significantly) with TM4SF5 expression in the liver (p = 0.1746), with NAS (p = 0.0522), and with BMI (p = 0.1447), in addition to insulin resistance and higher cholesterol levels in NAFLD patients.

Interestingly, we also found that the TM4SF5-dependent production of apelin could be linked to changes in SPP-1/OPN levels. SNU449-TM4SF5 cells expressed more apelin but lower SPP-1/OPN compared to control SNU449-EV cells. Exogenous apelin treatment of SNU449-EV and SNU449-TM4SF5 cells led to decreased and increased SPP1/OPN levels, respectively, in a dose-dependent manner, which was positively correlated with AKT1 and ELK1 activities. Indeed, AKT1 and ELK1 activities are involved in the expression of SPP1/OPN.^29^^,^^30^ A possible link between SPP1/OPN and obesity has been reported as CD153^+^PD-1^+^CD4^+^ T cells cause inflammation of visceral adipose tissue and insulin resistance under HFD conditions or in obesity by secreting SPP1/OPN.^31^ However, in this study, SPP1/OPN might be differentially functional for the regulation of food intake depending on TM4SF5 expression levels. With minimal or no TM4SF5 expression, apelin treatment gradually decreased SPP1/OPN expression, whereas apelin treatment of TM4SF5-positive hepatocytes caused increased SPP1/OPN levels in a dose-dependent manner. Furthermore, exogenous overexpression of TM4SF5 in hepatocytes or animals caused increased apelin but decreased SPP1/OPN levels, supporting an intrinsically antiparallel role between apelin and SPP1/OPN. Therefore, within complex liver tissues under chronic HFD_2w_ or HFD-DF_2w_ with or without hepatic TM4SF5 expression, the effect of SPP-1/OPN on food intake and BWG can be hardly explainable due to complicated influences by apelin, HFD, and/or DF. Nonetheless, the role of SPP1/OPN alone in food intake might be orexigenic or anorexigenic depending on TM4SF5 expression as we have not been able to analyze the effect of SPP-1/OPN alone without any influence by apelin in cell and animal studies.

This study also reveals NAFLD features even following HFD or HFD-DF for 1 or 2 weeks, especially in *Alb*-TG^Tm4sf5−Flag^ mice overexpressing TM4SF5 only in hepatocytes. For this, TM4SF5 supported food-intake behaviors for a greater amount or an abnormal eating pattern together with apelin expression for steatotic features. In addition, upon acute (less than a day) supply of fatty acids extracellularly to hepatocytes, TM4SF5 can bind fatty acid transporters, including solute carrier family 27 member 2 (SLC27A2) and member 5 (SLC27A5), to negatively regulate transporting activities.^32^ However, TM4SF5 plays positive roles in the chronic uptake and deposition of lipids into hepatocytes.^21^ Therefore, TM4SF5-positive hepatocytes chronically surrounded with fatty acids or lipids can accumulate them in the cells. Furthermore, TM4SF5 expression leads to increased chemokines, such as CCL20 and CXCL10, which in turn activate macrophages including Kupffer cells.^23^ Therefore, nutritional interventions or normally voluntary food-intake behaviors can be used to avoid NAFLD development,^33^ in addition to approaches that control the TM4SF5-mediated functions in immune-metabolic pathways. Therefore, the application of anti-TM4SF5 reagents or similar approaches may be promising ways to avoid the development of NAFLD, in addition to behavioral therapeutic approaches. Indeed, suppression of the TM4SF5 downstream effector CCL20 leads to the blockade of HFD-mediated NASH phenotypes,^23^ and *Tm4sf5*^−/−^ KO mice show protection from diet-induced obesity.^34^ Here, TM4SF5 expression led to ACOX1-mediated β-oxidation, concomitant mTOR activation, and autophagy inhibition. TM4SF5 in hepatocytes causes a pro-inflammatory hepatic environment, leading to macrophage activation and repolarization,^23^ which are positively involved in mTOR/S6K1 activation^19^ and hepatocellular carcinoma (HCC).^35^ Upon extracellular L-arginine supply, TM4SF5 in hepatocytes can translocate to lysosomal membranes to sense the physiological L-arginine inside lysosomes, leading to mTOR activation.^19^ mTOR activity inhibits autophagy.^36^ Interestingly, ACOX1-dependent peroxisomal β-oxidation is also involved in inflammatory cytokine production and the development of NASH pathology.^37^ Similar to the observations in this study, short-term obesogenic-diet challenge, such as a high-fat high-carbohydrate diet for 4 weeks, accelerates and exacerbates ACOX1-dependent peroxisomal β-oxidation-driven hepatocyte damage and systemic inflammation.^37^ Increased ACOX1 stabilizes PEX2 which in turn causes degradation of ATGL, leading to decrease in lipolysis.^27^ However, the TM4SF5-mediated effects appeared not to involve decreased lipolysis since TM4SF5 expression was not correlated with the PEX2 and ATGL expression levels. Thus, TM4SF5-mediated NAFLD-like phenotypes could be triggered following HFD or HFD-DF even for a short period of 1 or 2 weeks via promoted peroxisomal β-oxidation and concomitant mTOR activation and autophagy inhibition, in addition to a TM4SF5-mediated inflammatory environment. Longer HFD is still likely to lead to mitochondrial FAO and *de novo* lipogenesis (DNL) depending on hepatocyte TM4SF5 and may eventually cause NAFLD/NASH. Although we did not see dramatic immune cell infiltrations into the steatotic livers beyond the F4/80-positivity in immunohistology stains, but rather increased inflammatory chemokine expression levels together with hepatocyte damages and fat droplets, our study that has adopted HFD for 2 weeks could reveal TM4SF5-dependent features of earlier stages of NAFLD. Finally, this study provides evidence that TM4SF5 expression in hepatocytes can promote abnormal food-intake behaviors such as increased eating during inappropriate mealtimes, which is supported by TM4SF5-mediated apelin expression, leading to initiation of hepatic steatosis toward NAFLD features.

### Limitations of this study

Although we observed NAFLD features in mice during the short diet protocols performed in this study, observations of the infiltration of immune cells into the liver require more aggressive approaches to examine genetic profiles. In addition, apelin levels in diverse chronic liver diseases should be evaluated with greater patient sample numbers before statistical analyses of TM4SF5 and TM4SF5-related molecules and features.

## STAR★Methods

### Key resources table

**Table:** 

REAGENT or RESOURCE	SOURCE	IDENTIFIER
**Antibodies**		

Anti-α-tubulin (TU-02)	Santa Cruz Biotechnology	Cat# sc-8035, RRID:AB_628408
Anti-β-actin (C4)	Santa Cruz Biotechnology	Cat# sc-47778, RRID:AB_626632
Mouse anti-Osteopontin/SPP-1	R and D Systems	Cat# AF808, RRID:AB_2194992
Anti-apln/Apelin rabbit pAb	Abcam	Cat# ab125213, RRID:AB_10999708
Monoclonal anti-Flag M2 antibody	Sigma-Aldrich	Cat# F1804, RRID:AB_262044
Mouse anti-Akt1 mAb	Santa Cruz Biotechnology	Cat# sc-81434, RRID:AB_1118808
Anti-phospho-Akt1 (Ser473) rabbit pAb	Santa Cruz Biotechnology	Cat# sc-33437, RRID:AB_2225021
Anti-Elk1 rabbit pAb	Cell Signaling Technology	Cat# 9182, RRID:AB_2277936
Anti-phospho-Elk1 (Ser383) rabbit pAb	Thermo Fisher Scientific	Cat# PA5-17881, RRID:AB_10980224
Anti-Tm4sf5 (C-terminus) rabbit pAb	Homemade: Jung et al.^19^	https://pubmed.ncbi.nlm.nih.gov/36063136/
Anti-Srebp1 mouse mAb	Santa Cruz Biotechnology	Cat# sc-365513, RRID:AB_10843812
Anti-Srebp2 mouse mAb	Santa Cruz Biotechnology	Cat# sc-271616, RRID:AB_10708843
Anti-Fasn (C20G5) rabbit mAb	Cell Signaling Technology	Cat# 3180, RRID:AB_2100796
Anti-ACC rabbit mAb	Cell Signaling Technology	Cat# 33676, RRID:AB_2616592
Anti-phospho-ACC (Ser79) rabbit mAb	Cell Signaling Technology	Cat# 11818, RRID:AB_2687505
Anti-SCD1 (S-15) goat pAb	Santa Cruz Biotechnology	Cat# sc-14719, RRID:AB_656063
Anti-Dgat2 (C-15) goat pAb	Santa Cruz Biotechnology	Cat# sc-32400, RRID:AB_2090818
Anti-Acox1 rabbit pAb	Proteintech	Cat# 10957-1-AP, RRID:AB_2221670
Anti-LC3B rabbit pAb	Cell Signaling Technology	Cat# 2775, RRID:AB_915950
Anti-p62/SQSTM1 rabbit pAb	Cell Signaling Technology	Cat# 5114, RRID:AB_10624872
Anti-mTOR	Cell Signaling Technology	Cat# 2972, RRID:AB_330978
Anti-phospho-mTOR (Ser2448) (D9C2) rabbit mAb	Cell Signaling Technology	Cat# 5536, RRID:AB_10691552
Anti-phospho-mTOR (Ser2481) rabbit pAb	Cell Signaling Technology	Cat# 2974, RRID:AB_2262884
Anti-AMPK (D63G4) rabbit mAb	Cell Signaling Technology	Cat# 5832, RRID:AB_10624867
Anti-phospho-AMPKα (Thr172) (40H9) rabbit mAb	Cell Signaling Technology	Cat# 2535, RRID:AB_331250
Anti-Cpt1a (D3B3) rabbit mAb	Cell Signaling Technology	Cat# 12252, RRID:AB_2797857
Anti-Ulk1 (D8H5) rabbit mAb	Cell Signaling Technology	Cat# 8054, RRID:AB_11178668
Anti-phospho-Ulk1 (Ser555) rabbit mAb	Cell Signaling Technology	Cat# 5869, RRID:AB_10707365
phospho-Ulk1 (Ser757) pAb	Cell Signaling Technology	Cat# 6888, RRID:AB_10829226
Anti-Laminin-gamma-2 (E−6) mAb	Santa Cruz Biotechnology	Cat# sc-28330, RRID:AB_2134476
Anti-Col1a1 (H-197) rabbit pAb	Santa Cruz Biotechnology	Cat# sc-28657, RRID:AB_2229646
Anti-F4/80 (D2S9R) XP® Rabbit mAb	Cell Signaling Technology	Cat# 70076, RRID:AB_2799771
Anti-CCL2 mouse mAb (Clone T202)	Creative Diagnostics	Cat# CABT-35128MH, RRID:AB_2356195

**Bacterial and virus strains**		

Lentivirus for shRNAs against *TM4SF5*	This study	Table 2
siRNA against human *ELK1*	This study	Table 2

**Biological samples**		

High-fat diet, Teklad 60 kcal% fat	Orient. Co. Ltd, Seoul, Korea	58Y1

**Chemicals, peptides, and recombinant proteins**		

[pyr1]-apln/apelin-13 trifluoroacetate salt	Sigma-Aldrich	SML2084
4′-methoxy-4-dihydroxychalcone	Lee et al.^38^	https://www.ncbi.nlm.nih.gov/pubmed/28255353

**Critical commercial assays**		

Adiponectin Mouse ELISA kit	Abcam	ab108785
Mouse Ghrelin ELISA Kit	Cusabio	CSB-E09817m
Mouse GLP-1 ELISA Kit	Cusabio	CSB-E08118m
Mouse Glucagon ELISA Kit	Cusabio	CSB-E15775m
Mouse Leptin ELISA Kit	Abcam	ab100718
Immunohistochemistry-DAB staining, vectastain ABC-HRP kit	Vector Laboratories	PK-6100
Human/mouse [Pyr-1]-Apelin-13 EIA Kit	Phoenix Pharmaceuticals	EK-057-19
Human glucose ELISA kit	Abcam	Ab65333
Triglyceride ELISA kit	Bioassay Systems	ETGA-200
Cholesterol ELISA kit	Abcam	ab65390

**Deposited data**		

mRNA-Seq data from hepatocytes without or with TM4SF5	Sequence Read Archive (SRA) at NCBI	SRA accession number: PRJNA770813
Uncropped raw immunoblot gel images	Mendeley Data	https://data.mendeley.com/datasets/y9wwtjy74h/1

**Experimental models: Cell lines**		

SNU449 or SNU761 hepatocytes, TM4SF5-deficient	Lee et al.^39^	https://pubmed.ncbi.nlm.nih.gov/18357344/
HepG2 and Huh7 hepatocytes, endogenously TM4SF5-expressing	Lee et al.^39^	https://pubmed.ncbi.nlm.nih.gov/18357344/

**Experimental models: Organisms/strains**		

Mouse: Wild-type male C57BL/6NCrljOri	OrientBio, Seung-Nam, South Korea	N/A
Mouse: Tm4sf5-knockout (*Tm4sf5*^−/−^) male mice, C57BL/6	Jung et al.^19^	https://pubmed.ncbi.nlm.nih.gov/30956113/
Mouse: Hepatocyte-specific Tm4sf5 transgenic (*Alb*-TG^Tm4sf5−Flag^ male mice, C57BL/6	Jung et al.^20^	https://pubmed.ncbi.nlm.nih.gov/36063136/

**Oligonucleotides**		

siRNA and shRNA targeting sequences for XX, see Table 2	Cosmo Genetech (Seoul, Korea)	N/A
Primer sequences for XX, See Table 1	Cosmo Genetech (Seoul, Korea)	N/A

**Recombinant DNA**		

pCDNA3.1-FLAG	Jung et al.^19^	https://pubmed.ncbi.nlm.nih.gov/30956113/
TM4SF5-FLAG	Jung et al.^19^	https://pubmed.ncbi.nlm.nih.gov/30956113/

**Software and algorithms**		

GraphPad Prism 7	GraphPad software	N/A
NIS-Elements software	Nikon	N/A
Motic Digital Slide Assistant	MoticEasyScan, Motic	N/A

### Resource availability

#### Lead contact

Further information and requests for resources and reagents should be directed to and will be fulfilled by the Lead Contact, Jung Weon Lee (jwl@snu.ac.kr).

#### Materials availability

All newly generated plasmids and cell lines in this study are available by contacting the lead contact.

### Experimental model and study participant details

Experimental cell lines were purchased from Korean Cell Bank (Seoul, Korea) and ATCC (Manassas, VA, USA), and used for transient or stable transfection or infection processes with standard methods, as also described in the method details. Animal models with genetically altered *Tm4sf5* expression were previously reported in ref. ^19^^,^^20^ All animal procedures were performed in accordance with the procedures of the Seoul National University Laboratory Animal Maintenance Manual and with IRB approvals from the Institute of Laboratory Animal Resources, Seoul National University (SNU-200410-3 and SNU181016-7-4).

#### Cell lines

TM4SF5-deficient control human hepatocarcinoma SNU449 or SNU761 cells, TM4SF5-positive human hepatocytes, endogenously expressing HepG2 and Huh7 cells were used, as explained in the key resources table.

#### Animal models

Eight∼nine-week-old WT or *Tm4sf5*^-/-^ male C57BL/6 mice (n = 4 per group) were used for diet animal models; Animals were fed *ad libitum* access for either an entire day or daytime (from 9:00 am to 9:00 pm) only with NCD (NCD_1w_ or NCD-DF_1w_) or HFD (HFD_1 or 2w_ or HFD-DF_1 or 2w_) or access either *ad libitum* or restricted daytime feeding with HFD (HFD_2w_ or HFD-DF_2w_) together with the injection of either saline or ^pyr1^apelin-13 (Sigma-Aldrich, 300 μg/kg body weight) intraperitoneally. All animal procedures were conducted in compliance with protocols from the Seoul National University (SNU) Laboratory Animal Maintenance Manual and were approved by the Institutional Review Board of the Institute of Laboratory Animal Resources Seoul National University (SNU-200410-3 and SNU181016-7-4).

### Method details

#### Cells

HepG2, SNU449, and Huh7 hepatocytes were obtained from either the Korean Cell Bank (Seoul, Korea) or ATCC (Manassas, VA). The hepatocytes were regularly evaluated and found negative for mycoplasma. Cells were maintained under standard conditions in humidified incubators at 37°C and 5% CO_2_ in either high-glucose Dulbecco’s Modified Eagle Medium (DMEM) or Roswell Park Memorial Institute (RPMI) medium (Hyclone, Logan, UT) supplemented with 10% fetal bovine serum (FBS; GenDEPOT, Barker, TX) and 5% penicillin/streptomycin (GenDEPOT). Every 3-4 days, the cells were passaged at 80-90% confluency. The human hepatocarcinoma cell line SNU449 was stably transfected with empty vector control (EV) or TM4SF5 cDNA plasmids. Endogenously TM4SF5-expressing cell lines such as HepG2 and Huh7 were infected or transfected with shRNA and siRNA, respectively. The siRNA or cDNA plasmids were transfected for 48 h using Lipofectamine RNAiMAX or Lipofectamine 3000 (Thermo Fisher Scientific, Waltham, MA), respectively, according to the manufacturer’s protocols. For stable cell lines, cells were infected with lentivirus for shRNA against a non-specific (NS) or TM4SF5 sequence (listed in Table 2) for 24 h, using vector pLKO.1 (Addgene). Infected cells were selected using puromycin (2 μg/ml, GenDEPOT).

#### Cell culture and apelin treatment

Cells were seeded in 6-well plates to 60% confluency in either high-glucose DMEM or RPMI medium supplemented with 10% FBS and 5% penicillin/streptomycin. Cells were cultured in serum-free media. After 24 h, treatment with ^pyr1^Apelin-13 at different doses (0, 1, 5, 8, 10, and 100 nM, Sigma-Aldrich) was performed for 24 h or indicated periods. *In vitro* cell experiments were performed at least three independent times, and each experiment was performed in triplicate.

#### Cell transfection

Stably expressing SNU449 cells with either empty vector (EV) or TM4SF5 expression vector were prepared and seeded at a density of 1x10^5^ cells/cm^2^ in 60-mm cell plates in complete RPMI (supplemented with 10% FBS and 5% penicillin/streptomycin antibiotics). The cells were incubated in a 5% CO_2_ incubator at 37°C. When the confluency reached 60-80 percent, siRNA against the control sequence or target sequence against *TM4SF5* or *ELK1* (see Table 2) was transfected with using Lipofectamine™ RNAiMax. Cells were incubated in a 5% CO_2_ incubator at 37°C for the transgene expression for 24-72 h before harvesting.

#### Mice

Male WT, *Tm4sf5* knockout (*Tm4sf5*^-/-^ KO), and hepatocyte-specific *TM4SF5-*overexpressing (via the albumin promoter conjugated to the Flag-tagged mouse *Tm4sf5* gene [*Alb*-TG^Tm4sf5-Flag^]) transgenic (TG) C57BL/6 mice were used for the *in vivo* experiments. TG^Tm4sf5-Flag^ and systemic KO mice were developed as described in a previous report.^19^ All animal procedures were conducted in compliance with protocols from the Seoul National University (SNU) Laboratory Animal Maintenance Manual and were approved by the Institutional Review Board of the Institute of Laboratory Animal Resources Seoul National University (SNU-200410-3 and SNU181016-7-4). Male mice were housed at 22-24°C with a 12 h light/dark cycle. After weaning, animals were preferentially housed in groups of 3-4 littermates in ventilated cages prior to specific diet/peptide treatment. Animals had *ad libitum* access to water and a standard NCD or HFD (60% fat kCal, D12492, ENVIGO) before experiments or treatments.

#### Mice 1-week diet experiment

Eight-week-old male C57BL/6 WT, TG, and KO mice were acclimatized to the facility for 7 days prior to the experiment. At 9 weeks old, mice were fed with *ad libitum* access for either an entire day or daytime only with NCD (NCD_1w_ or NCD-DF_1w_) or HFD (HFD_1w_ or HFD-DF_1w_) for one week. Restricted daytime-only feeding lasted from 9:00 am to 9:00 pm every day. Body weights were measured at 9:00 am every day.

#### Mice 2-week diet and ^pyr^apelin-13 injection experiment

Nine-week-old male C57BL/6 WT, TG, and KO mice had access either *ad libitum* or restricted daytime feeding with HFD (HFD_2w_ or HFD-DF_2w_) together with the injection of either saline or ^pyr1^apelin-13 (Sigma-Aldrich, 300 μg/kg body weight) intraperitoneally. Mice were injected once daily following body weight measurement in the morning (9:00 am), prior to the start of feeding for HFD-DF groups. ^pyr1^Apelin-13 was dissolved in 1× sterile PBS and aliquoted before the fresh preparation of an injected dose.

#### Body weight and food intake analyses

Age-matched animals were randomly grouped for the experimental conditions described above. During the diet periods, the body weights of mice were measured every day at 9:00 am and graphed as mean ± standard deviation (SD) values. Food intake was also measured by giving the same food amount every day, and the remaining food was measured at two different time points (around 8:30-9:00 am and 8:30-9:00 pm). At the end of the animal experiments, body weights and liver weights were measured before sacrificing at 9:00 am. In addition to collecting blood samples, the largest lobules of the liver were collected in cassettes for tissue staining, and the other lobules were immediately frozen in cryotubes containing RNA-later and stored at -80°C until use.

#### Human NAFLD serum and liver tissue analyses

The stored samples of serum and liver parenchyma of patients with NAFLD were analyzed. The patients were enrolled in another study at Ewha Womans University Mokdong Hospital (approval no.: EUMC 2016–07–052; approval date: 8-30-2016). Written informed consent was obtained from each participant including permission for the use of stored samples for secondary analysis with another purpose. Histopathological analysis was performed on specimens from percutaneous liver biopsies. Ultrasonography-guided liver biopsies were performed by expert radiologists or hepatologists with experienced from more than 200 liver biopsies. A transthoracic approach was routinely used with the patient in the supine position. Two specimens were obtained from each patient to acquire a sample of sufficient size for analysis and to reduce histologic errors. Each liver tissue sample was analyzed by 2 experienced pathologists who were blinded to the patients’ clinical information. The minimum adequacy of the specimen was defined as a length longer than 2 cm.^40^ Liver fibrosis was scored according to the Nonalcoholic steatohepatitis Clinical Research Network scoring system for NAFLD and the METAVIR scoring system for other etiologies, ranging from F0 to F4.^41^ Each sample was scored according to the NAFLD activity score (NAS).^41^ Lobular inflammation and steatosis were scored on a 0-3 scale, and ballooning and portal inflammation were scored on a 0-2 scale. The fatty liver inhibition of progression algorithm was used to define the presence of NASH.^42^

#### Plasma biochemical analyses

Whole animal or human blood samples were separated by being allowed to sit for 30 min or using EDTA-coated tubes, followed by centrifugation at 4000 ×*g* for 15 min and storage at -80°C until use. Blood glucose, triglycerides, total cholesterol, glucose, and AST/ALT were measured using Fuji Dri-Chem Slides (Fujifilm). Leptin, ghrelin, GLP-1, adiponectin, and glucagon concentrations were determined in duplicate according to the manufacturer’s ELISA instructions. ELISA kits for mouse adiponectin and leptin were purchased from Abcam (ab108785 and ab100718; Cambridge, UK), and ELISA kits for mouse ghrelin, GLP-1, and glucagon were from Cusabio (CSB-E09817m, CSB-E08118m, and CSB-E15775m, respectively; Houston, TX). Enzyme immunoassay (EIA) kits for human/mouse apelin-13 were purchased from Phoenix Pharmaceutical (EK-057-19, Burlingame, CA). ELISA kits for glucose (Ab6533, Abcam), triglycerides (ETGA-200, Bioassay Systems, Hayward, CA, USA), and cholesterol (ab65390, Abcam) were used to measure levels in the liver tissues and sera saved after the experimental diets and from control and NAFLD patients.

#### Hematoxylin and Eosin (H&E) staining

The right lobe of the liver was fixed in 10% neutral buffered formalin and embedded in paraffin. Seven-micrometer-thick sections were deparaffinized and rehydrated before staining with H&E.^21^ Histologically stained liver tissues were scanned and randomly saved using MoticEasyScan (Motic, British Columbia, Canada) and Motic DSAssistant software.

#### Western blots

Proteins were extracted from frozen mouse or human liver or cells in RIPA lysis buffer containing 1% Brij 58 and proteinase inhibitors (P3100, GenDEPOT). Samples were homogenized for 2 min and centrifuged for 15 min at 12000 ×*g*. Protein concentrations were measured using Pierce™ BCA Protein Assay Kit (Thermo Fischer Scientific) and normalized, before the addition of 6× sample buffer and standard western blots. The primary antibodies we used in this study are listed in Key Resources Table; most were used at a 1:1,000 dilution ratio in TBS-T containing 5% BSA. Subsequently, membranes were washed three times (each for 10 min) with TBS-T buffer and incubated with the corresponding horseradish peroxidase (HRP)-conjugated secondary antibody: HRP goat anti-rabbit 1:5,000 or HRP goat anti-mouse 1:3,000 diluted in 1× TBST-T containing 5% skim milk, for 1 h at RT.

#### qRT-PCR

The piece of liver (approximately 10-20 mg) for each animal was cut and put in cryotubes containing RNA-later, then stored at -80°C. Total RNA from cells or mouse liver tissue samples was prepared and analyzed with qRT-PCR, as explained previously.^19^ Primers are listed in Table 1. Quantification was done using the delta Ct method and normalized to the control group in each experiment. GAPDH was used for normalization within each sample.

#### Analysis of public RNA expression data

mRNA expression levels were searched in the public Gene Expression Omnibus (GEO; https://www.ncbi.nlm.nih.gov/geo/) database (accession numbers GSE48325), as explained previously.^35^

### Quantification and statistical analysis

Data are presented as mean ± SD. Statistical analyses were performed using Prism Software (GraphPad 7.0, La Jolla, CA, USA). The unpaired two-tailed Student’s *t*-test and one-way or two-way ANOVA were performed depending on the analysis to determine statistical significance. A *p* value ≤ 0.05 was considered statistically significant. ∗, ∗∗, ∗∗∗, and ∗∗∗∗ indicate *P* ≤ 0.05, *P* ≤ 0.01, *P* ≤ 0.001, and *P* ≤ 0.0001 respectively. ns indicates non-significance.

## Data Availability

• The RNA-seq raw datasets used in this study have been deposited into the Sequence Read Archive (SRA) database at NCBI. SRA accession number is PRJNA770813. Original (uncropped) western blot images have been deposited at Mendeley and are publicly available as of the date of publication. Microscopy and quantitative (q)RT-PCR data reported in this paper are shared by the lead contact upon request. • This study did not generate any novel software or algorithms. Published and freely available software and algorithms used for analysis in this study are listed in the key resources table. Original codes are shared by the lead contact upon request. • Any additional information required to reanalyze the data reported in this paper is available from the lead contact upon request.
