# Supplementary material for: TM4SF5-mediated abnormal food-intake behavior and apelin expression facilitate non-alcoholic fatty liver disease features
Source: iScience. 2023 Aug 14;26(9):107625. doi: 10.1016/j.isci.2023.107625 (PMC10475478; doi:10.1016/j.isci.2023.107625)
Supplement: Document S1. Figures S1–S4 [file mmc1.pdf]

iScience, Volume *26*

Supplemental information

TM4SF5-mediated abnormal food-intake behavior and apelin expression facilitate non-alcoholic fatty liver disease features

Yangie Dwi Pinanga, Han Ah Lee, Eun-Ae Shin, Haesong Lee, Kyung-hee Pyo, Ji Eon Kim, Eun Hae Lee, Wonsik Kim, Soyeon Kim, Hwi Young Kim, and Jung Weon Lee

Figure S1

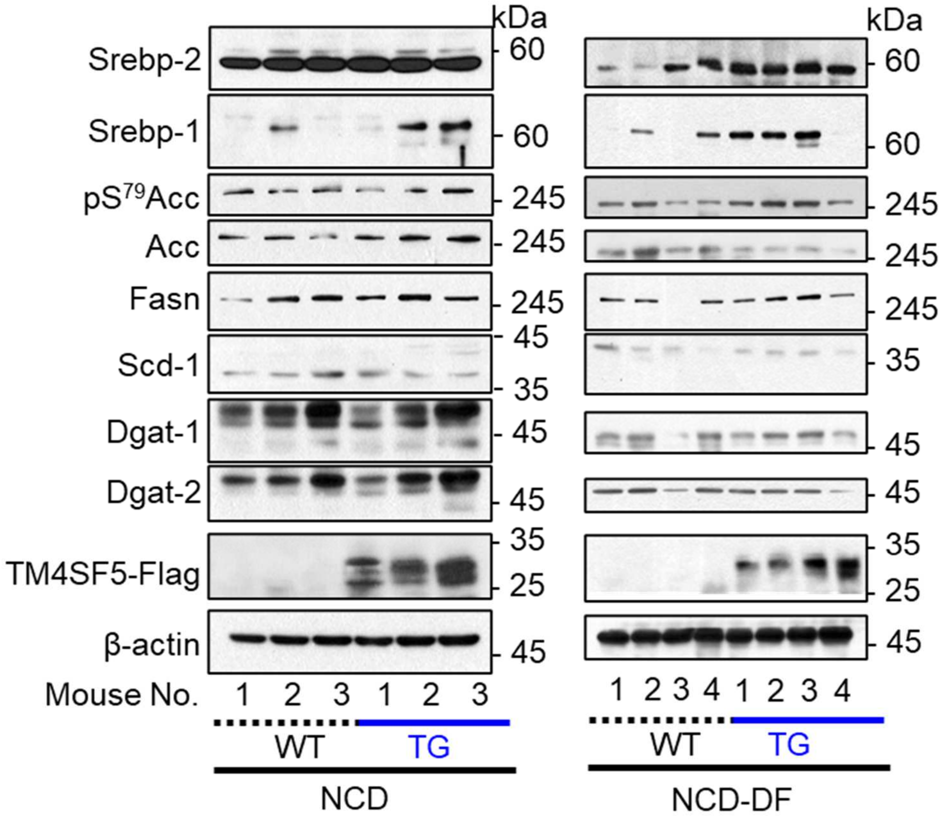

Fig. S1. Expression of lipogenic molecules in liver tissues of WT or *Alb*-TG$^{Tm4sf5\text{-}Flag}$ mice fed NCD$_{1w}$ or NCD-DF$_{1w}$. Liver tissues were collected from male 3-month-old C57BL/6 WT or *Alb*-TG$^{Tm4sf5\text{-}Flag}$ (TG) mice before whole tissue extract preparation and immune blots for the indicated molecules. The TM4SF5-Flag immunoblot in the left panel has been recycled from Fig. 1I, since samples from the same experimental conditions were used. Related to Figure 1.

Figure S2

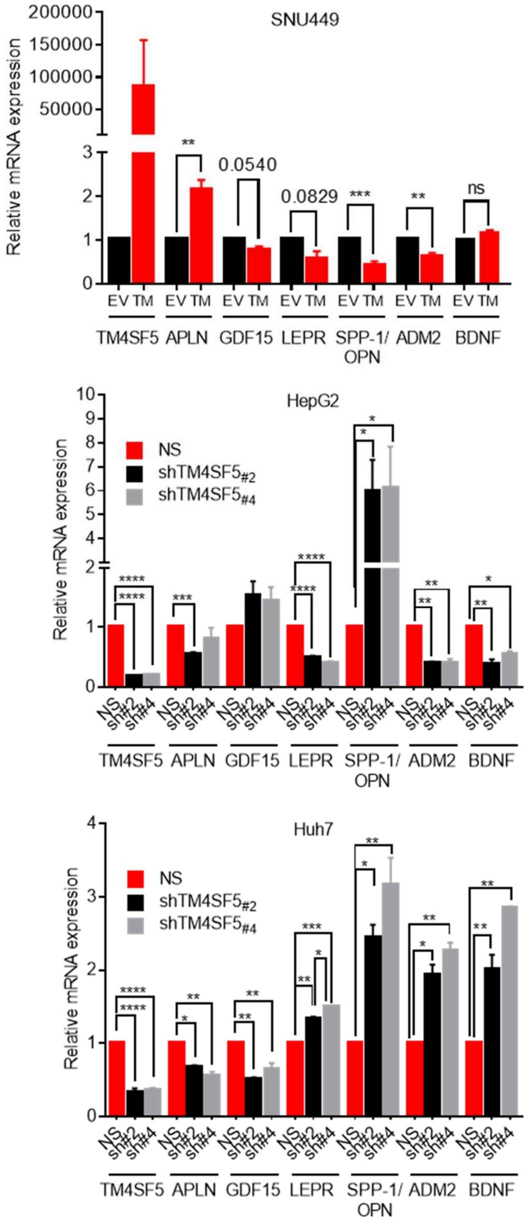

Fig. S2. Inverse relation between *Apln* and *Spp1* mRNA levels in *in vitro* overexpression or suppression cell systems. Stable SNU449 cell lines expressing empty vector (EV) or TM4SF5 (TM) (Top), or HepG2 or Huh7 cells transfected with control shRNA against non-specific sequence (NS) or shTM4SF5 against TM4SF5 sequences (#2 or #4, see Table 1) were processed for qRT-PCR for the indicated molecules. Primers are listed in Table 2. *p*-values were calculated by the Dunnett's test and ordinary one-way ANOVA. *, **, ***, or **** depict statistical significance at $p<0.05$, 0.01, 0.001, or 0.0001, respectively. ns depicts non-significance. Data represent three independent experiments. Related to Figure 2.

Figure S3.

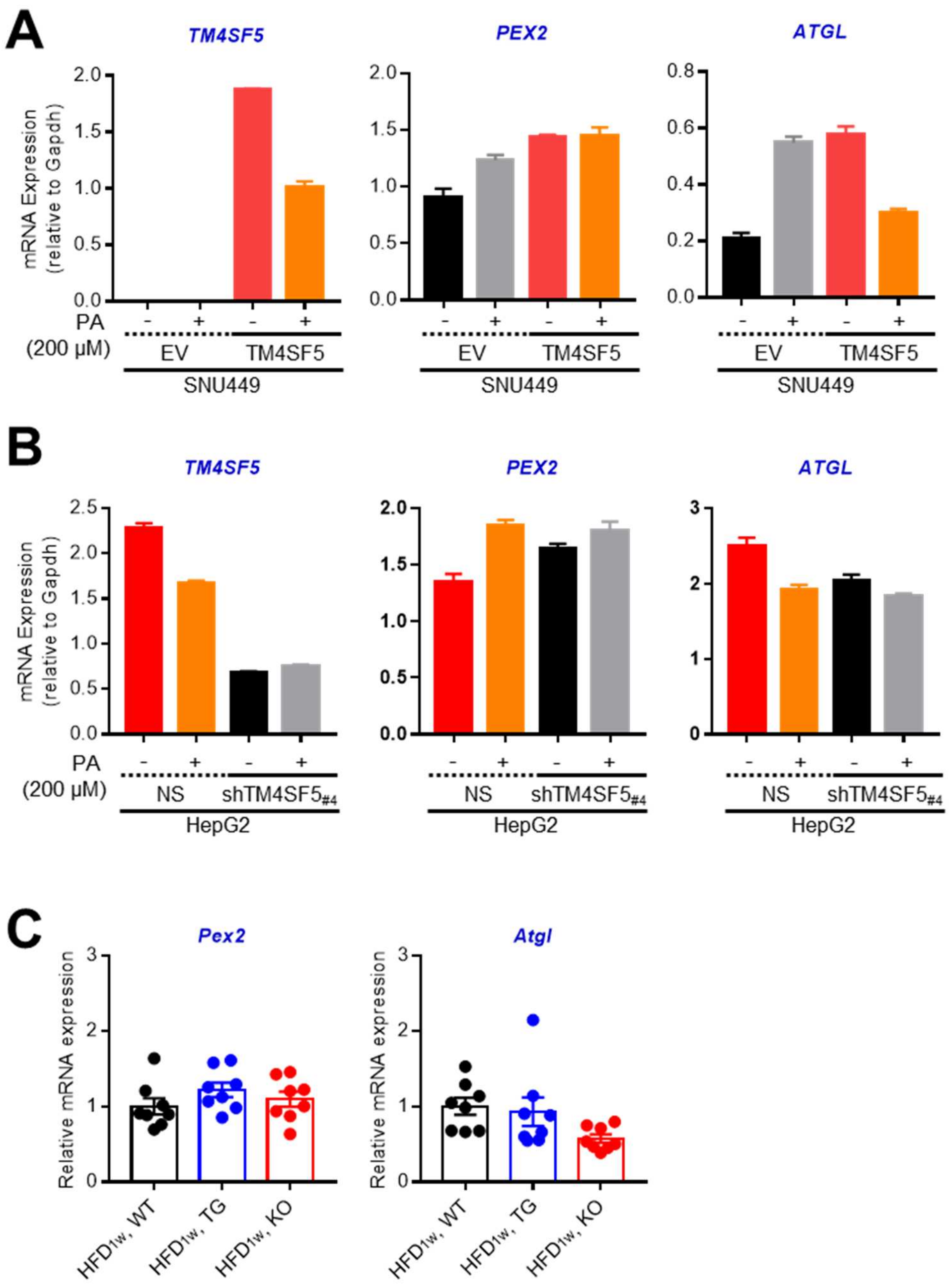

Fig. S3. TM4SF5 expression was not correlated with levels of the PEX2-ATGL linkage. (A and B) Stable SNU449-EV or SNU449-TM4SF5 cells (A) or HepG2 cells (B) with shRNA against non-specific sequence (shNS) or against TM4SF5 sequence (shTM4SF5$_{\#4}$, see Table 1) were treated with palmitic acid (PA, 200 μM for 24 h), before qRT-PCR analysis for *TM4SF5*, *PEX2*, and *ATGL*. **(C)** Liver tissues from WT, TG, or KO mice fed HFD$_{1w}$ were analyzed for *Pex2* or *Atgl* mRNA levels. Data shown represent three independent experiments. Related to Figure 6.

Figure S4.

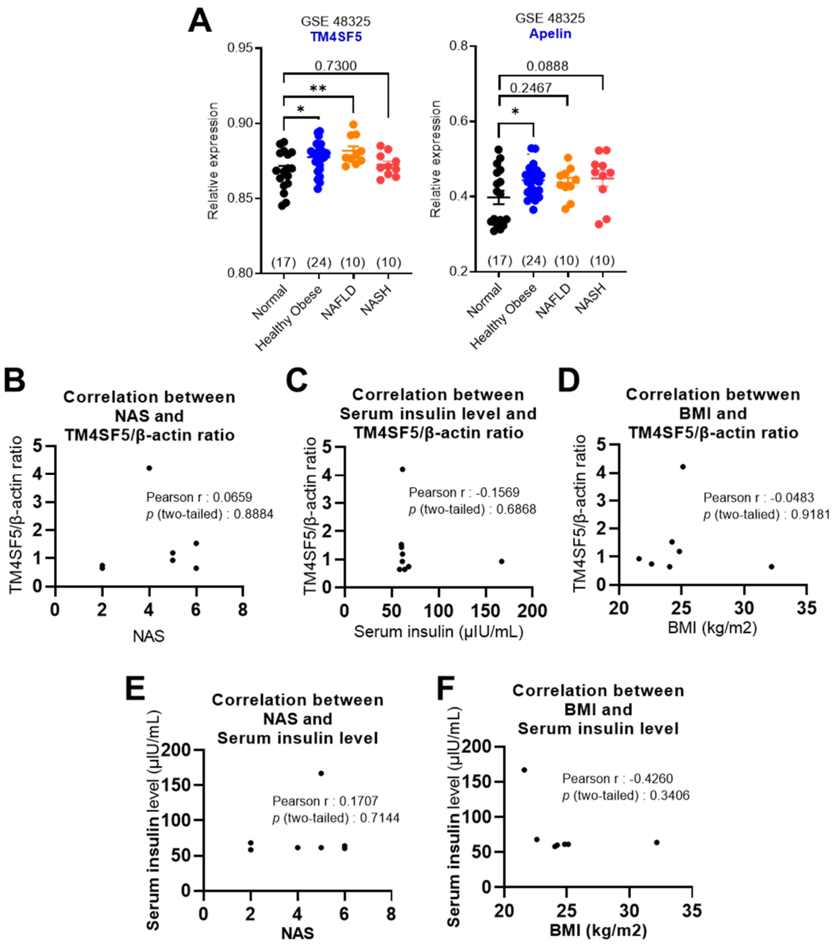

Figure S4. Analyses on associations among TM4SF5 expression, serum insulin, BMI, or NAS in non-NAFLD individuals or NAFLD patients. (A) TM4SF5 or Apelin expression profiles of NAFLD [normal vs healthy obese vs NAFLD vs NASH] from GSE48325 were analyzed. *p*-values were calculated by the non-paired student *t* test or Dunnett's test and ordinary one-way ANOVA. *, $P < 0.05$; **, $P < 0.01$. **(B to F)** Seven serum and liver tissue sample pairs from non-NAFLD (n=2), NASH-Fibrosis (n=4), and NASH-Cirrhosis (n=1) human (A to E) individuals or patients were analyzed for ELISA or immunoblots. TM4SF5 expression in livers were measured by standard immunoblots where the band intensity was normalized with that of β-actin. Note that TM4SF5 expression alone did not correlate with serum insulin level in the samples, and either parameter was not correlated with NAS or BMI, either. Meanwhile, serum insulin level was negatively correlated with TM4SF5 expression or BMI, although their correlations were not statistically significant. Related to Figure 8.
